# Diabetes mellitus and the risk of gastrointestinal cancer in women compared with men: a meta-analysis of cohort studies

**DOI:** 10.1186/s12885-018-4351-4

**Published:** 2018-04-16

**Authors:** Hong-juan FANG, Shao-bo SHAN, Yu-hao ZHOU, Li-yong ZHONG

**Affiliations:** 1 Department of Endocrinology, Beijing Tiantan Hospital, Capital Medical University, 6 Tiantan Xili, Dongcheng District, Beijing, 100050 China; 2 Department of Neurosurgery, Beijing Tiantan Hospital, Capital Medical University, 6 Tiantan Xili, Dongcheng District, Beijing, 100050 China; 3 Department of Rehabilitation Institute, Seventh People’s Hospital of Shanghai University of TCM, Datong road 358, Pudong District, Shanghai, 200137 China

**Keywords:** Diabetes mellitus, Gastrointestinal cancer, Sex difference, Meta-analysis

## Abstract

**Background:**

The increasing epidemic proportions of diabetes mellitus (DM) are a major cause of premature illness and death. However, whether DM confers the same excess risk of gastrointestinal cancer for women as it does for men remains controversial. The purpose of this study was to estimate the relation between DM and gastrointestinal cancer in women compared with men after accounting for other major risk factors based on cohort studies.

**Methods:**

We performed a meta-analysis of cohort studies published through May 2017 from PubMed, Embase, and the Cochrane Library. Studies with cohort designs were stratified by sex and reported the relation between DM and esophageal cancer (EC), gastric cancer (GC), colorectal cancer (CRC), colon cancer (CC), rectal cancer (RC), hepatocellular carcinoma (HCC), or pancreatic cancer (PC) risk. The ratio of relative risk (RRR) between men and women was employed to measure the sex differences in the relation between DM and gastrointestinal cancer with a random effects model with inverse variance weighting.

**Results:**

We included 38 cohort studies reporting data on 18,060,698 individuals. The pooled RRR indicated DM women was associated with an increased risk of GC (RRR: 1.14; 95%CI: 1.06–1.22; *p* < 0.001), while the risk of HCC was lower (RRR: 0.88; 95%CI: 0.79–0.99; *p* = 0.031) as compared with DM men. Further, there was no evidence of sex differences in the RRR between participants who had DM compared with those without DM for EC (*p* = 0.068), CRC (*p* = 0.618), and PC (*p* = 0.976). In addition, the pooled RRR showed a statistically significant association between DM and the risk of CC in women compared with men (RRR: 0.93; 95%CI: 0.86–1.00; *p* = 0.050), and there was no evidence of sex differences for RC among participants with DM compared to those without DM (*p* = 0.648). Finally, the sex differences of the comparison between DM and non-DM for gastrointestinal cancer risk at different sites were variable after stratification for different effect estimates.

**Conclusions:**

The findings of this study suggested female-to-male RRR of DM was increased for GC, while reduced for HCC and CC. However, there were no sex differences for the relation between DM and the risk of EC, CRC, PC, and RC.

**Electronic supplementary material:**

The online version of this article (10.1186/s12885-018-4351-4) contains supplementary material, which is available to authorized users.

## Background

Diabetes mellitus (DM) is a growing global pandemic afflicting approximately three to 4 % of adults worldwide; it has caused around 1.3 million deaths worldwide [1, 2]. The International Diabetes Federation (IDF) indicated that 387 million people throughout the world had DM in 2014; this may rise to 592 million by 2035 [3]. Prevalence of DM is a risk factor for cardiovascular disease, blindness, kidney failure, amputations, fractures, cognitive impairment, and several cancers [4–15]. In addition, women with DM have a significantly greater risk of lung and renal cell cancer, non-Hodgkin’s lymphoma, and myeloma than do men with DM [16–18]. Whether this sex difference exists for DM and gastrointestinal cancer including esophageal cancer (EC), gastric cancer (GC), colorectal cancer (CRC), colon cancer (CC), rectal cancer (RC), hepatocellular carcinoma (HCC), or pancreatic cancer (PC) remains debatable.

In 2004, the Cancer Prevention Study II reported that women with DM had a 38% greater reduction of HCC risk than did their male counterparts [19, 20]. Further, the National Health Screening Service indicated DM was associated with higher risk of CRC in women, while this association had no significant difference in men [21]. Conversely, Adami et al. found DM played a harmful effect on RC in men, while it had no significant effect in women [22]. In addition, Wang et al. found significant increased standard incidence ratios for EC, GC, HCC, or PC were observed in women with DM than in men [23]. Lin et al. demonstrated increased cancer risks in participants with type 2 DM and suggested women had higher risk of GC and lower PC risk when compared with men [24]. Wideroff et al. suggested excess colon and HCC risk in men than women with DM [25]. Fedeli et al. indicated men with DM had greater HCC risk than did women [26]. However, these studies compared DM with the general population and reported that standard incidence/mortality ratios (SIR/SMR) might contribute biases when compared with relative risk (RR), odds ratio (OR), or hazard ratio (HR). Estimates of the sex-specific relation between DM and subsequent EC, GC, CRC, CC, RC, HCC, PC risk were not illustrated in previous meta-analyses; this was because direct comparisons of the relation between DM and gastrointestinal cancer in men and women were not performed within-study comparisons in each of the studies [10–15]. Clarifying the sex difference of DM and gastrointestinal cancer risk is particularly important as it has not been definitively determined. Here we attempt a large-scale examination of the available cohort studies that reported sex-specific effects of DM on subsequent risk of gastrointestinal cancer including EC, GC, CRC, CC, RC, HCC, and PC.

## Methods

### Data sources, search strategy, and selection criteria

This study was conducted and reported according to the meta-analysis of observational studies in epidemiology protocol [27]. Any cohort study that examined the relation between DM and gastrointestinal cancer including EC, GC, CRC, CC, RC, HCC, and PC risk written in the English language was eligible for inclusion in our study, and there were no restrictions based on publication status (published, in press, or in progress). We searched the PubMed, Embase, and Cochrane Library electronic databases for articles published through May 2017 and used (“diabetes mellitus” OR “diabetes”) AND (“cancer” OR “carcinoma” OR “neoplasm” OR “tumour”) AND “cohort” AND “human” AND “English” as the search terms. We also conducted manual searches of reference lists from all the relevant original and review articles to identify additional eligible studies. The medical subject heading, methods, patient population, design, exposure, and outcome variables of these articles were used to identify the relevant studies.

The literature search and study selection were conducted by two authors independently using a standardized approach. Any inconsistencies between these two authors were settled by the primary author until a consensus was reached. A study was eligible for inclusion if the following criteria were met: (1) the study had to have a cohort design; (2) the study investigated the association between DM and the risk of gastrointestinal cancer including EC, GC, CRC, CC, RC, HCC, and PC; (3) the study reported the association between DM and gastrointestinal cancer in men and women simultaneously; and (4) the authors reported effect estimates (SIR, SMR, RR, OR, or HR) and 95% confidence intervals (CIs) for comparisons of DM and non-DM. Studies performed on single-sex populations were excluded. Further, we excluded all case-control studies because various confounding factors could bias the results.

### Data collection and quality assessment

The data collection and quality assessment were conducted by two authors independently. Information was examined and adjudicated by an additional author referring to the original studies independently. The data collected included the first author or study group’s name, publication year, country, study design, sample size, mean age or age range, number of men and women, type of DM, percentage of smokers, reported outcomes, effect estimate, duration of follow-up, and maximum adjustment. For studies that reported several multivariable adjusted effect estimates, we selected the effect estimate that was maximally adjusted for potential confounders. The study quality was evaluated by the Newcastle-Ottawa Scale (NOS), which is quite comprehensive and has been partially validated for evaluating the quality of observational studies [28]. Further, the methodological quality was based on selection (4 items), comparability (1 item), and outcome (3 items). A “star system” (range, 0–9) has been developed for assessment (Additional file 1).

### Statistical analysis

We examined the relation between DM and risk of gastrointestinal cancer on the basis of the sex-specific effect estimate and its 95% CI published in each study. HR was considered equivalent to RR in cohort studies. Given the low incidence of gastrointestinal cancer, OR could be assumed to be accurate estimates of RR. We first used the random-effects model to calculate summary RRs and 95% CIs for DM versus non-DM and the risk of EC, GC, CRC, CC, RC, HCC, and PC in men and women separately [29, 30]. Then, sex-specific RRs and 95%CIs were employed to estimate the female-to-male ratio of RRs (RRR) and 95%CIs in the individual study (Additional file 2) [31, 32]. The random-effects model were employed to calculate pooled RRRs and 95%CIs for the sex difference of DM versus non-DM and the risk of gastrointestinal cancer (EC, GC, CRC, CC, RC, HCC, and PC) [29, 30]. Further, the results of SIR/SMR and RR/OR/HR for sex difference of relation between DM and gastrointestinal cancer were combined separately due to SIR/SMR reported cancer risk according to compared DM patients with general population, and the comparability are differ with RR/OR/HR. Heterogeneity between studies was investigated by using the I^2^ and Q statistic, and we considered *p* values < 0.10 as indicative of significant heterogeneity [33, 34]. A sensitivity analysis was performed to investigate the influence of a single study in meta-analysis on the overall risk estimates by excluding one by one sequentially [35]. Meta-regression analysis was conducted for EC, GC, CRC, CC, RC, HCC, and PC to investigate the impact of publication year, sample size, mean age, percentage of smokers and follow-up duration on data heterogeneity [36]. Subgroup analyses were conducted on the basis of publication year (2010 or after, before 2010), country (Eastern, Western), study design (prospective, retrospective), sample size (≥100,000, < 100,000), mean age (≥60, < 60), DM types (Type 1, Type 2, both), follow-up duration (≥10, < 10), adjusted body mass index (BMI) (yes, no), adjusted smoking (yes, no), adjusted alcohol intake (yes, no), and adjusted physical activity (PA) (yes, no). Visual inspections of funnel plots for EC, GC, CRC, CC, RC, HCC, and PC were conducted. At the same time, Egger’s [37] and Begg’ s test [38] were employed to quantitatively evaluate potential publication bias. The significant level (α) were 0.05 for all pooled analyses. Statistical analyses were performed using STATA software (version 10.0; Stata Corporation, College Station, TX, USA).

## Results

### Literature search

**Fig. 1:**
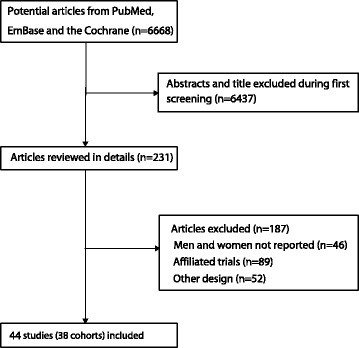
Flow diagram of the literature search and studies selection process

The results of the study-selection process are shown in Fig. 1. We initially identified 6668 potentially eligible articles after the original electronic search. Of these, 6437 articles were excluded during an initial review of titles and abstracts. Full texts for the remaining 231 articles were retrieved to identify potential included studies, and 44 studies reported 38 cohorts that satisfied the inclusion criteria, which ultimately were included in the meta-analysis [19–26, 39–74]. A manual search of the reference lists contained within these studies did not yield any new eligible studies. The general characteristics of the included studies are presented in Table 1 and the effect estimate in each study is shown in Additional file 3.

**Table 1:** Baseline characteristic of studies included in the systematic review and meta-analysis

Study	Country	Study design	Sample size	Mean age or age range	Men/women	DM types	Percentage of smoker (%)	Reported outcomes	Effect estimate	Follow-up duration (years)	Maximum adjustment
Verona Diabetes Study 2003 [38]	Italy	Prospective	7148	66.6	3366/3782	II	20.9	EC death, GC death, CRC death, HCC death, PC death	SMR	10.0	Age, treatment, smoking, BMI
Veneto Region 2014 [26]	Italy	Prospective	167,621	30.0–89.0	91,521/76,100	II	NA	EC death, GC death, CRC death, HCC death, PC death	SMR	3.0	Age, specific regional
Uppsala Health Care Region 1991 [22]	Sweden	Prospective	51,008	> 20.0	27,862/23,146	Both	NA	EC, GC, CC, RC, HCC, PC	RR	19.0	Age, follow-up duration
Takayama Study cohort 2013 [39]	Japan	Prospective	28,565	55.2	14,173/16,547	Both	32.7	EC, GC, CC, RC, HCC, PC	HR	13.2	Age at baseline, smoking, BMI, PA, education, history of hypertension, stroke, IHD, TEI, and intake of fat, ethanol, and coffee
The Singapore Chinese Health Study 2006/2013 [40, 41]	Singapore	Prospective	61,320	56.4	27,328/33,992	Both	30.6	CRC, HCC	HR	10.0	Age, year of recruitment, gender, dialect group, education, smoking, alcohol, BMI, and consumption of coffee and tea
SMHS and SWHS 2013/2015 [42, 43]	China	Prospective	133,288	53.8	61,491/74,942	II	33.0	GC, HCC	HR	5.5/11.2	Age, birth cohort, education, income, BMI, occupation, smoking, alcohol, family history of cancer, TEI, fruit intake, vegetable intake, total PA, history of CH or CLD, HRT, and menopausal status
Ragozzino 1982 [44]	US	Prospective	1135	NA	NA	Both	NA	GC, CRC, PC	RR	8.6	Age
Limburg 2006 [45]	US	Retrospective	1975	61.0	997/978	II	57.0	CRC	SIR	16.9	Age and calendar period
PHARMO Database 2017 [46]	Netherlands	Prospective	68,076	63.9	34,686/33,390	II	NA	EC, GC, CC, RC, HCC, PC	HR	13.0	Age, use of statins, PPI, anti-hypertensives 90 days prior to start of each time-interval
Gini 2016 [47]	Italy	Retrospective	32,247	65.0	17,827/14,420	II	NA	CRC, HCC, PC	SIR	3.7	Age and year at cancer diagnosis
NIH-AARP Diet and Health Study 2011 [48]	US	Prospective	469,448	62.0	280,883/188,565	Both	14.0	EC, GC	HR	8.0	Age, calories, alcohol, smoking, fruit consumption, vegetable consumption, ethnicity, education, and PA
Korean Cancer Prevention Study 2005 [49]	Korea	Prospective	1,298,385	46.9	829,770/468,615	II	38.9	GC death, CRC death, HCC death, PC death	HR	10.0	Age, age squared, smoking, and alcohol
NHANESI 1995 [50]	US	Prospective	14,407	49.1	NA	Both	30.2	CRC	OR	17.0	Age, BMI, smoking, alcohol, income, and recreational PA
The Cardiovascular Health Study 1999 [51]	US	Prospective	5849	72.8	2478/3371	Both	12.0	CRC	RR	6.4	Age and PA
Fujino 2001 [52]	Japan	Prospective	4902	55.2	2444/2458	Both	30.3	HCC	RR	10.3	Age, smoking, alcohol, history of hepatitis, and cirrhosis
Clalit Health Care Services 2013 [53]	Israel	Retrospective	87,934	51.5	43,632/44,302	II	5.8	CRC, PC	SIR	10.0	Age
Clalit Health Services 2016 [54]	Israel	Prospective	2,186,196	46.6	1,034,074/1,152,122	II	31.6	GC, CRC, HCC, PC	HR	10.1	Age, socioeconomic status, and ethnic group
Danish Central Hospital Discharge Register 1997 [25]	Denmark	Prospective	109,581	66.5	54,571/55,010	Both	NA	EC, GC, CC, RC, HCC, PC	SIR	5.7	Age, follow-up duration
Cancer Prevention Study 1998 [55]	US	Prospective	863,699	52.3	352,849/510,850	Both	40.6	CRC	RR	13.0	Age, race, educational level, BMI, smoking, alcohol, dietary intake, aspirin use, PA, and family history of CRC
D2C cohort 2011 [56]	Germany	Prospective	26,742	64.0	12,650/14,092	II	17.2	CRC, HCC, PC	SIR	3.7	BMI, diabetes duration, and medication at study entry
Diabetes Registry Tyrol 2014 [57]	Austria	Prospective	7627	59.0	4126/3501	Both	NA	GC, CRC, HCC, PC	SIR	6.0	Age
Koskinen 1998 [58]	Finland	Prospective	114,058	30.0–74.0	NA	Both	NA	GC, CC	SMR	5.0	Age
EPIC-Norfolk Study 2004 [59]	UK	Prospective	9605	59.0	4445/5160	Both	11.7	CRC	RR	6.0	Age, BMI, and smoking
Xu 2015 [60]	China	Retrospective	36,379	59.0	16,166/20,213	II	NA	GC, CC, RC, HCC, PC	SIR	3.7	Age
Newfoundland and Labrador 2013 [61]	Canada	Retrospective	122,228	> 30.0	61,156/61,072	Both	NA	CRC, CC, RC	HR	10.5	Age, and severity of co-morbid illness
Netherlands Cohort Study 2016 [62]	Netherlands	Prospective	120,852	55.0–69.0	58,279/62,573	II	NA	CRC, CC, RC	HR	17.3	Age, BMI, pants/skirt size, family history of CRC, smoking, alcohol, dietary habits and nonoccupational PA
Maccabi Healthcare Services 2010 [63]	Israel	Retrospective	100,595	61.6	52,913/47,682	Both	9.6	EC, GC, CC, RC, HCC, PC	HR	8.0	Age, region, SES level, use of healthcare services a year prior to index date, BMI, and history of CVD
National Health Screening Service 2001 [21]	Norway	Prospective	75,219	49.2	36,975/38,244	Both	NA	CRC, CC	RR	12.0	Age
Nationwide Cohort Study in Sweden 1995 [64]	Sweden	Retrospective	134,096	NA	63,987/70,109	Both	NA	PC	SIR	6.7	Age entered cohort, year entered cohort, initial hospitalization, hospital discharges, diabetic complications, follow-up
The Multiethnic Cohort 2010 [65]	US	Prospective	199,142	59.9	89,478/109,664	Both	NA	CRC	RR	13.0	Race, age at baseline questionnaire, BMI, smoking, NSAIDs use, education, alcohol, saturated fat intake, unsaturated fat intake, dietary fibre intake, PA, family history of CRC.
Wang 2015 [23]	China	Prospective	327,268	59.4	163,819/163,449	II	NA	EC, GC, CC, RC, HCC, PC	SIR	7.0	Age and urbanization level of area registered in the system
Zhang 2012 [66, 67]	China	Retrospective	7938	61.1	3792/4146	II	NA	EC, GC, CRC, CC, RC, HCC, PC	SIR	2.6	Age, surveillance region and the observed person-years in type 2 diabetes
Japan Public Health Center- Based Prospective Study 2006 [68, 69]	Japan	Prospective	97,771	51.6	46,548/51,223	Both	40.8	GC, CC, RC, HCC, PC	HR	10.7	Age at baseline, study area, history of cerebrovascular disease, history of IHD, smoking, ethanol intake, BMI, leisure-time PA, green vegetable intake, and coffee intake
Cancer Prevention Study II 2004 [19, 20]	US	Prospective	1,056,243	56.7	467,922/588,321	Both	20.3	EC death, GC death, CC death, RC death, HCC death, PC death	RR	14.8	Age, race, years of education, BMI, smoking, alcohol, total red meat consumption, consumption of citrus fruits and juices, consumption of vegetables, PA
Japan Collaborative Cohort Study 2006 [70, 71]	Japan	Prospective	56,881	57.1	23,378/33,503	Both	23.5	GC, CRC death, CC, RC, HCC, PC	RR	8.0	Age, BMI, family history of cancer, smoking, drinking habit, sports, walking, and education
National Health Insurance Program 2014 [24]	China	Retrospective	9,884,228	> 20.0	5,419,238/4,464,990	II	NA	EC, GC, CRC, HCC, PC	SIR	10.0	Insurance premium, urbanization degree of area registered for National Health Insurance program, and age
Zhou 2010 [72]	7 countries in Europe	Prospective	44,655	53.3	26,460/18,195	Both	34.1	HCC death, PC death	HR	5.9–36.8	Study cohort, age, BMI, SBP, cholesterol and smoking
EPOCH-JAPAN 2017 [73]	Japan	Prospective	46,387	57.7	20,426/25,961	Both	27.5	PC death	HR	14.6	Age, BMI, smoking, and alcohol

BMI: body mass index; CC: colon cancer; CH: chronic hepatitis; CLD: chronic liver disease; CRC: colorectal cancer; CVD: cardiovascular disease; DM: diabetes mellitus; EC: esophagus cancer; GC: gastric cancer; HCC: hepatocellular carcinoma; HR: hazard ratio; HRT: hormone replacement therapy; IHD: ischemic heart disease; OR: odds ratios; PA: physical activity; PC: pancreatic cancer; PPI: proton pump inhibitors; RC: rectal cancer; RR: relative risk; SBP: systolic blood pressure; SIR: standardized incidence ratio; SMHS: Shanghai Men’s Health Study; SMR: standardized mortality ratio; SWHS: Shanghai Women’s Health Study; TEI: total energy intake; UK: United Kingdom; US: Unite States

### Study characteristics

Of the 38 cohorts involving a total of 18,060,698 individuals, 29 were prospective cohort designs [19–23, 25, 26, 39–45, 47, 49–53, 55–60, 63, 66, 69–74] and the remaining 9 were retrospective cohort designs [24, 45, 48, 54, 61, 62, 64, 65, 67, 68]. The follow-up period for participants was 2.6–36.8 years, while 1135–9,884,228 individuals were included in each trial. Fourteen cohorts were conducted in Europe [21, 22, 25, 26, 39, 47, 48, 57–60, 63, 65, 73], 15 in Asia [23, 24, 40–44, 50, 53–55, 61, 64, 67–72, 74], and 9 in the US or Canada [19, 20, 45, 46, 49, 51, 52, 56, 62, 66]. Further, 15 of the included cohorts reported the relation between Type 2 DM and the risk of gastrointestinal cancer [23, 24, 26, 39, 43, 44, 46–48, 50, 54, 55, 57, 61, 63, 67, 68], while the remaining 23 cohorts included both Type 1 and Type 2 DM [19–22, 25, 40–42, 45, 49, 51–53, 56, 58–60, 62, 64–66, 69–74]. The study reported EC available in 11 cohorts [19, 20, 22–26, 39–47, 49, 64, 67, 68], GC in 21 cohorts [19, 20, 22–26, 39, 40, 43–45, 47, 49, 50, 55, 58, 59, 61, 64, 67–72], CRC in 22 cohorts [21, 24, 26, 39, 41, 42, 45, 46, 48, 50–52, 54–58, 60, 62, 63, 66–68, 71, 72], CC in 15 cohorts [19, 23, 25, 40–47, 59, 61–64, 67–72], RC in 13 cohorts [19, 20, 22, 23, 25, 40, 47, 61–64, 67–72], HCC in 22 cohorts [19, 20, 22–26, 39–44, 47, 48, 50, 53, 55, 57, 58, 61, 64, 67–73], and PC in 24 cohorts [19, 20, 22–26, 39, 40, 45, 47, 48, 50, 54, 55, 57, 58, 61, 64, 65, 67–74]. The SIR/SMR were employed to measure the strength of associations in 14 cohorts [23–26, 39, 46, 48, 54, 57–59, 61, 65, 67, 68], and the remaining 24 studies used RR/OR/HR as an effect measure index [19–22, 40–45, 47, 49–53, 55, 56, 60, 62–64, 66, 69–74]. One article included data from 17 European population-based or occupational cohorts [73]^,^ and another article combined 10 cohorts in Japan [74]. Study quality was evaluated using the NOS (Additional file 1). Overall, 16 cohorts had a score of 9 [19–22, 40–42, 47, 50, 51, 53, 55, 56, 62, 63, 66, 69, 70, 74], 8 had a score of 8 [43–45, 49, 52, 60, 64, 71–73], 4 had a score of 7 [24, 39, 46, 54], and the remaining 10 had a score of 6 [23, 25, 26, 48, 57–59, 61, 65, 67, 68].

### DM and gastrointestinal cancer risk in men and women separately

The summary RRs for gastrointestinal cancer at different sites were calculated and presented in Additional file 4. First, there was no significant difference between DM and EC risk in men and women whether based on SIR/SMR or RR/OR/HR. Second, women with DM were associated with an increased risk of GC based on SIR/SMR, while there was no significant effect according to RR/OR/HR. Further, DM was not associated with the risk of GC in men. Third, both men and women participants with DM were associated with increased risk of CRC and CC whether based on SIR/SMR or RR/OR/HR, whereas only men with DM was associated with an increased risk of RC based on SIR/SMR. Fourth, both men and women with DM were associated with greater risk of HCC on the basis of SIR/SMR or RR/OR/HR. Finally, men and women with DM were correlated with greater risk of PC based on SIR/SMR or RR/OR/HR.

### Esophagus cancer

**Fig. 2:**
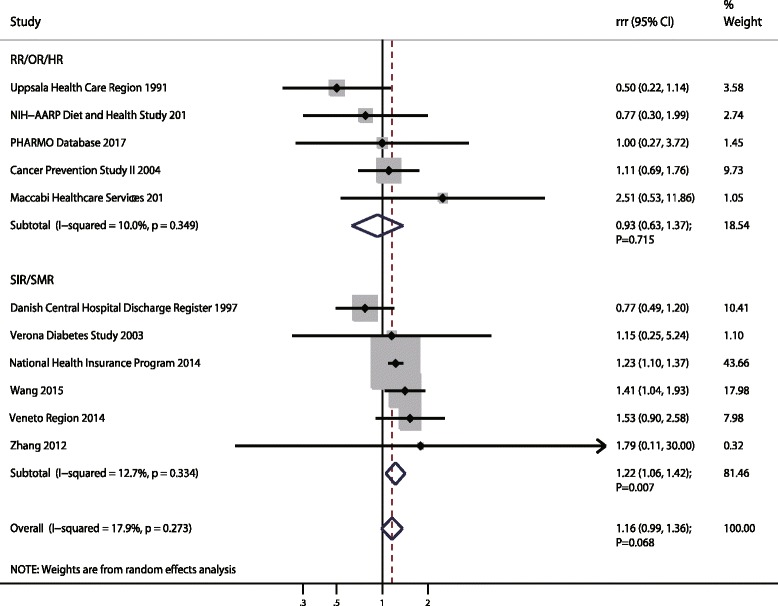
The female-to-male ratio of relative risk for esophagus cancer, diabetes mellitus compared with non-diabetes mellitus

A total of 11 cohorts in 16 studies reported an association between DM and subsequent EC risk [19, 20, 22–26, 39, 40, 47, 49, 64, 67, 68]. The pooled RRR (female to male) of DM versus non-DM was 1.16 (95%CI: 0.99–1.36; *p* = 0.068; Fig. 2); this was not statistically significant and there was no evidence of between study heterogeneity (I^2^ = 17.9%; *p* = 0.273). Further, the pooled RRR (female to male) based on SIR/SMR for the risk of EC was increased (RRR: 1.12; 95%CI: 1.06–1.42; *p* = 0.007), while this sex difference based on RR/OR/HR was not associated with statistically significant (RRR: 0.93; 95%CI: 0.63–1.37; *p* = 0.715). Sensitivity analysis suggested no sex difference between DM and EC based on SIR/SMR when excluding the study conducted by Wang et al. [23], a study specifically with a large sample size and reported lower incidence of EC in men (Additional file 5). Meta-regression analyses based on publication year, sample size, mean age, percentage of smokers and follow-up duration were conducted and the results showed these factors were not associated with a sex difference of DM and EC risk (Additional file 6). Subgroup analyses conducted for EC were separated by SIR/SMR and RR/OR/HR. When based on SIR/SMR, we noted DM women were associated with greater risk of EC than DM men if the study was published in 2010 or after, conducted in Eastern countries, had a retrospective design, a mean age < 60.0 years, patients with Type 2 DM, follow-up duration ≥10.0 years, and was not adjusted for BMI, smoking, alcohol, or PA, respectively (Additional file 7). Finally, there were no sex differences for the relation between DM and EC risk according to pre-defined factors when data were pooled from RR/OR/HR. A publication bias was conducted and suggested no significant publication biases were detected for EC (*p* value for Egger: 0.452; *p* value for Begg: 0.755; Additional file 8).

### Gastric cancer

**Fig. 3:**
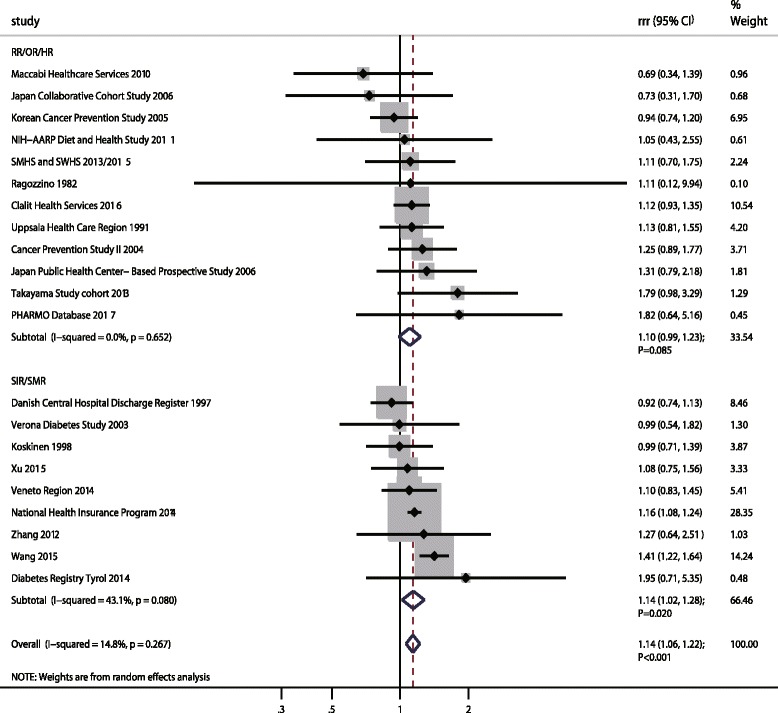
The female-to-male ratio of relative risk for gastric cancer, diabetes mellitus compared with non-diabetes mellitus

A total of 21 cohorts in 26 studies reported an association between DM and subsequent GC risk [19, 20, 22–26, 39, 40, 43–45, 47, 49, 50, 55, 58, 59, 61, 64, 67–72]. The pooled RRR (female to male) suggested that DM women was associated with an increased risk of GC as compared with DM men (RRR: 1.14; 95%CI: 1.06–1.22; *p* < 0.001; Fig. 3), and unimportant heterogeneity was observed (I^2^ = 14.8%; *p* = 0.267). This sex difference was detected when the result based on SIR/SMR (RRR: 1.14; 95%CI: 1.02–1.28; *p* = 0.020). Further, although women with DM had a 1o% higher risk of GC than men with DM, this sex difference was not statistically significant when combined results from RR/OR/HR (RRR: 1.10; 95%CI: 0.99–1.23; *p* = 0.085). The findings of sensitivity analyses suggested the sex difference was statistically significant based on SIR/SMR when excluding the National Health Insurance Program study [24], which specifically included a large sample size and contributed highly weighted in pooled results (Additional file 5). Further, when the summary results were based on RR/OR/HR, we noted the pooled RRR showed a statistically significant association between DM and the risk of GC in women when compared with men. Meta-regression analysis was conducted according to different effect estimate, and we detected publication year and mean age might have contributed to the association between DM and GC in women compared with men on the basis of SIR/SMR; other factors were not significant contributors to the association between DM and GC risk in women compared with men (Additional file 6). Subgroup analyses pooled SIR/SMR suggested the RRR (female to male) of DM was increased in GC if the study was conducted in 2010 or later, in Eastern countries, and used a retrospective design; further associations were mean age < 60.0 years, patients with Type 2 DM, follow-up duration ≥10.0 years, and no adjustment for BMI, smoking, alcohol, or PA, respectively (Additional file 7). Additionally, we noted that women with DM were associated with greater risk of GC than in men with DM if the follow-up duration was ≥10.0 years according to pooled analysis of RR/OR/HR. Finally, there was no publication biases for GC (*p* value for Egger: 0.664; *p* value for Begg: 0.415; Additional file 8).

### Colorectal cancer, colon cancer, and rectal cancer

**Fig. 4:**
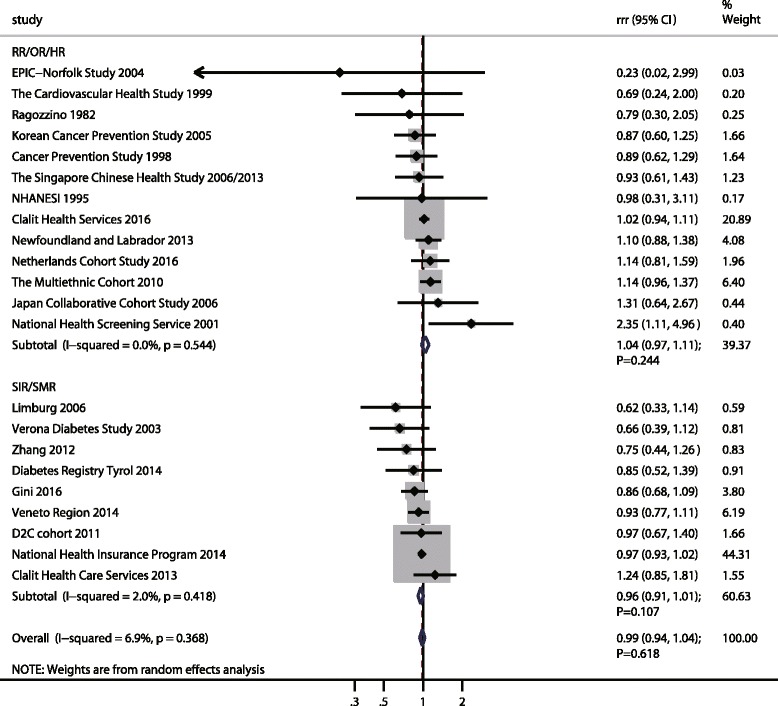
The female-to-male ratio of relative risk for colorectal cancer, diabetes mellitus compared with non-diabetes mellitus

A total of 22 cohorts in 25 studies reported an association between DM and subsequent CRC risk [21, 24, 26, 39, 41, 42, 45, 46, 48, 50–52, 54–58, 60, 62, 63, 66–68, 71, 72]. There was no evidence of a sex difference in the RR for CRC among participants with DM compared to those without DM (RRR: 0.99; 95%CI: 0.94–1.04; *p* = 0.618; Fig. 4), and unimportant heterogeneity across included studies was noted (I^2^ = 6.9%; *p* = 0.368). This conclusion was consistent whether the summary results were based on SIR/SMR (RRR: 0.96; 95%CI: 0.91–1.01; *p* = 0.107) or RR/OR/HR (RRR: 1.04; 95%CI: 0.97–1.11; *p* = 0.244). Sensitivity analysis was performed and the conclusion was not affected after the sequential exclusion of each study from all the pooled analyses (Additional file 5). Meta-regression analyses found there were no factors contributing to the association between DM and CRC risk in women compared with men (Additional file 6). The findings of subgroup analyses indicated the pooled RRR of DM versus non-DM and CRC risk in women was lower than in men if the study was conducted before 2010, in Western countries, and with a mean age ≥ 60.0 years based on SIR/SMR (Additional file 7). No significant publication bias was observed (*p* value for Egger: 0.609; *p* value for Begg: 0.367; Additional file 8).

**Fig. 5:**
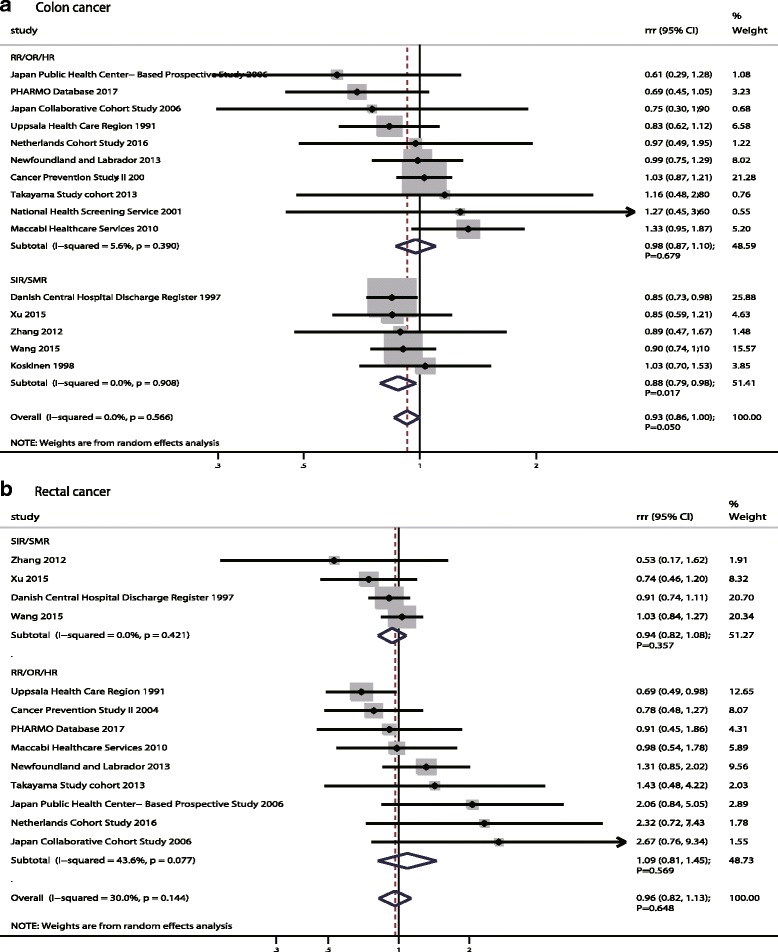
The female-to-male ratio of relative risk for colon cancer (**a**) and rectal cancer (**b**), diabetes mellitus compared with non-diabetes mellitus

Similarly, the pooled RRR (female to male) of DM versus non-DM for CC was 0.93 (95%CI: 0.86–1.00; *p* = 0.050; Fig. 5a) with no evidence of between-study heterogeneity (I^2^ = 0.0%; *p* = 0.566). Further, this significant sex difference mostly came from pooled SIR/SMR data (RRR: 0.88; 95%CI: 0.79–0.98; *p* = 0.017), and there was no sex difference when combined the study reported RR/OR/HR (RRR: 0.98; 95%CI: 0.87–1.10; *p* = 0.679). The findings of sensitivity indicated no sex difference based on SIR/SMR when excluding the Danish Central Hospital Discharge Register study [25]; this study specifically contributed highly weighted in pooled results (Additional file 5). The findings of meta-regression analysis suggested no factors played a significant effect on the relation between DM and CC in women compared with men (Additional file 6). When stratified for the sex difference based on the study reported SIR/SMR, we noted the risk of CC was significantly lower in women with DM as compared with DM men if the study was published before 2010 and conducted in Western countries; in addition, this lower significance held if the study had a prospective design, sample size ≥100,000, mean age ≥ 60.0 years, patients with both Type 1 and Type 2 DM, follow-up duration < 10.0 years, and was not adjusted for BMI, smoking, alcohol, or PA, respectively. Further, women with DM might have had a lower risk of CC as compared with men if the pooled study sample size was < 100,000 according to study reported RR/OR/HR (Additional file 7). Finally, there was no publication bias among included studies (*p* value for Egger: 0.982; *p* value for Begg: 0.767; Additional file 8).

In pooled data from 13 cohorts in 17 studies [19, 20, 22, 23, 25, 40, 47, 61–64, 67–72], there was no evidence of a sex difference for RC between participants with DM compared with those without DM (RRR: 0.96; 95%CI: 0.82–1.13; *p* = 0.648; Fig. 5b), and no significant heterogeneity was reported (I^2^ = 30.0%; *p* = 0.144). This conclusion was not changed when pooled with SIR/SMR (RRR: 0.94; 95%CI: 0.82–1.08; *p* = 0.357) and RR/OR/HR (RRR: 1.09; 95%CI: 0.81–1.45; *p* = 0.569), respectively. A sensitivity analysis was conducted and after each study was sequentially excluded from the pooled analysis; the conclusion was not affected by the exclusion of any specific study (Additional file 5). Further, we noted publication year and follow-up duration might affect the sex difference for DM and RC risk by meta-regression (Additional file 6). Similarly, no sex difference for the relation between DM and the risk of RC were detected in subgroup analyses (Additional file 7). Finally, there was no significant publication bias detected (*p* value for Egger: 0.285; *p* value for Begg: 0.161; Additional file 8).

### Hepatocellular carcinoma

**Fig. 6:**
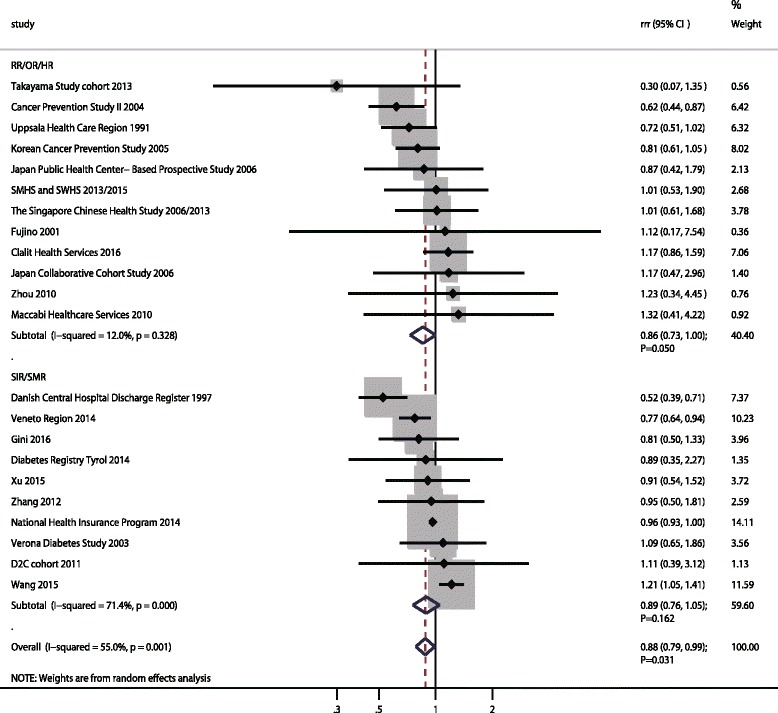
The female-to-male ratio of relative risk for hepatocellular carcinoma, diabetes mellitus compared with non-diabetes mellitus

A total of 22 cohorts in 29 studies reported an association between DM and subsequent HCC risk [19, 20, 22–26, 39–44, 47, 48, 50, 53, 55, 57, 58, 61, 64, 67–73]. The pooled RRR (female to male) of DM versus non-DM on HCC risk was 0.88 (95%CI: 0.79–0.99; *p* = 0.031; Fig. 6), and significant heterogeneity was detected (I^2^ = 55.0%; *p* = 0.001). This significant sex difference existed if based on pooled RR/OR/HR (RRR: 0.86; 95%CI: 0.73–1.00; *p* = 0.050), while no sex difference was based on pooled SIR/SMR (RRR: 0.89; 95%CI: 0.76–1.05; *p* = 0.162). The findings of sensitivity analysis suggested these sex differences were variable after sequentially excluding a single study, which might be because the power was not strong enough; this should be verified in future studies (Additional file 5). Further, publication year might have affected this sex difference by using meta-regression (Additional file 6). Subgroup analyses suggested women with DM had a lower HCC risk as compared with men with DM if the study was conducted in Western countries, and had a retrospective design, mean age < 60.0 years, patients with both Type 1 and Type 2 DM, and follow-up duration ≥10.0 years according to pooled SIR/SMR data. Besides, the pooled RRR (female to male) was reduced when the study was published before 2010 and conducted in Western countries, and if the study had a prospective design, patients with both Type 1 and Type 2 DM, and was adjusted for smoking, alcohol, and PA, respectively according to the results from RR/OR/HR (Additional file 7). The publication bias test results showed there was no evidence of publication bias (*p* value for Egger: 0.299; *p* value for Begg: 0.463; Additional file 8).

### Pancreatic cancer

**Fig. 7:**
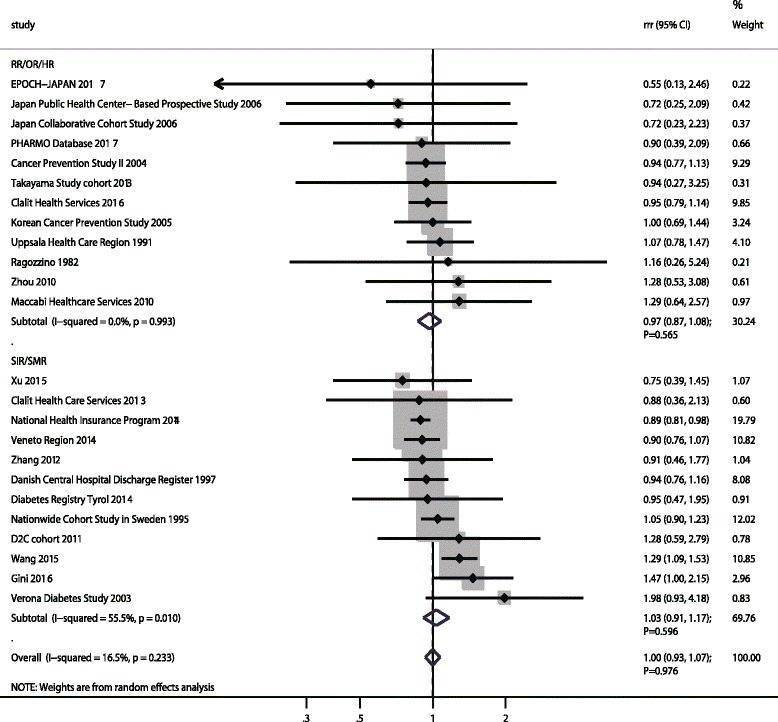
The female-to-male ratio of relative risk for pancreatic cancer, diabetes mellitus compared with non-diabetes mellitus

A total of 24 cohorts in 28 studies reported an association between DM and subsequent PC risk [19, 20, 22–26, 39, 40, 45, 47, 48, 50, 54, 55, 57, 58, 61, 64, 65, 67–74]. The pooled RRR indicated no sex difference for PC risk between participants with DM and those without DM (RRR: 1.00; 95%CI: 0.93–1.07; *p* = 0.976; Fig. 7), and with unimportant heterogeneity among included studies (I^2^ = 16.5%; *p* = 0.233). This insignificant sex difference persisted whether pooled with SIR/SMR data (RRR: 1.03; 95%CI: 0.91–1.17; *p* = 0.596) or RR/OR/HR data (RRR: 0.97; 95%CI: 0.87–1.08; *p* = 0.565). The conclusion was not affected after sequential exclusion of each study from the pooled analyses (Additional file 5). Further, we noted publication year, sample size, mean age, percentage of smokers and follow-up duration did not affect the sex difference of the relation between DM and PC risk (Additional file 6). In addition, this insignificant sex difference was stable and unchanged when stratified by pre-defined factors (Additional file 7). Finally, publication bias test results showed no evidence of publication bias (p value for Egger: 0.363; *p* value for Begg: 0.941; Additional file 8).

## Discussion

Over the past decades, the prevalence of DM has increased worldwide and has been especially remarkable in Asian countries [1, 75]. Previous studies have already demonstrated positive associations between DM and certain site-specific cancers, including liver, pancreas, endometrium, colon/rectum, breast, and bladder [76]. Whether the association of DM with cancer risk differs between men and women remains unclear. In the present study, a meta-analysis on the included cohort studies was conducted to explore correlations of all possible sex differences between DM and the incidence of gastrointestinal cancer. Further, sensitivity and subgroup analyses were performed to evaluate these sex differences among specific subpopulations. This comprehensive, quantitative study included 18,060,698 individuals from 38 cohort studies. The findings of our study suggested that in women with DM was associated with an increased risk of GC, while reduced risk of HCC and CC than in DM men as compared with those without DM. Further, the findings of sensitivity and subgroup analyses suggested that sex differences might exist for the associations between DM and EC or CRC risk.

As compared with previous meta-analyses, this study was a first meta-analysis to directly compare the sex difference of associations of DM with gastrointestinal cancer risk. Pang et al. conducted a meta-analysis of 22 cohort studies and found DM patients with a 52% excess risk of PC as compared those without DM [10]. Guraya et al. indicated DM women was associated with an increased risk of CRC, and the risk increased by 22% and 17% for women and men respectively [12]. The study conducted by Shimoyama et al. suggested DM patients with a greater magnitude risk of GC in females (RR: 1.90) than in males (RR: 1.24) [14]. Huang et al. found the risk of EC was increased in DM men, while this increased was not observed in DM women [15]. The inherent limitation of that previous meta-analyses included evidence level and these associations in patients with specific characteristics were not illustrated. Further, direct comparisons of sex differences for the relations between DM and gastrointestinal cancer were not calculated. We therefore performed this meta-analysis of available cohort studies to evaluate the relation between DM and gastrointestinal cancer in women compared with men.

The sex differences for the associations of DM and gastrointestinal cancer may be exhibited through several underlying mechanisms. First, DM patients with hyperglycemia leads to oxidative stress, which could promotes the formation and expression of advanced glycation products and their receptor. The interaction effect of advanced glycation products and their receptor with oxidative stress could active numerous cell signaling pathways, which could promote carcinogenesis and cell invasion [77, 78]. Second, through multiple cellular signaling cascades, enhanced insulin and insulin-like growth factor could promote cell proliferation and growth [79, 80]. Finally, the sex differences of the associations of DM and gastrointestinal cancer could attributable to various sex hormone-binding globulin, which could affect the bioavailability of estrogen in both sexes and bioavailable testosterone in women [81].

The findings of this study might have affected the true RR difference between the sexes for several reasons. First, at different age stages, the prevalence of DM differed between men and women. In our study, the age in each study was older than 20.0, while the mean age in most of the included studies ranged from 46.6 to 72.8 years. This factor might affect the balance of the DM and the non-DM groups and bias the pooled results [1]. Second, duration of DM is an important determinant of DM-related gastrointestinal cancer risk, and might thus have an effect on the sex ratio of RRs. Although the data was abstracted through whole cohorts in individual studies, several of the included studies have already illustrated a different effect estimate after excluding participants with short-term DM [48, 53, 55, 69, 71]. Finally, the cancer incidence between men and women was variable, which could affect the sex difference of the relation between DM and cancer at different sites [82]. Ultimately, considering the potential uncontrollable biases, we critically analysed our recommendations for the sex difference of the relation between DM and gastrointestinal cancer.

There was no significant sex difference between DM and non-DM and the risk of EC, CRC, RC, and PC. However, DM women showed greater risk of GC and lower risk of CC and HCC than DM men. In the summary analyses according to different effect estimates, we noted women with DM with greater risk of EC (SIR/SMR) and GC (SIR/SMR), while with reduced risk of CC (SIR/SMR) and HCC (RR/OR/HR) than in men with DM. The several possible reasons follow. (1) SIR and SMR were employed as an effect measure in 14 cohorts, and the general population were regarded as a non-exposed control; the comparability of characteristics was inferior to selection representativeness of cohorts as control group. (2) The small number of included cohorts might affect the sex difference of DM and EC risk. (3) Women with DM showed higher incidence of GC, while the prevalence of DM in Asian women was lower than in men, and the duration of DM was less. (4) Different compare control groups might play an important role in the progression of CRC. (5) The sex difference of CC was determined in individual studies, which contributed an important role and accounted for higher weight [19, 25]. (6) No sex difference of RC existed, and the sex difference of the relation between DM and CRC was attributed to CC. (7) The sex difference of DM and HCC risk might be confounded by hepatitis B virus infection; the majority of included studies did not adjust for this factor. (8) The prevalence of PC was lower than expected, and the correlates of DM and PC were stronger [10]; the true sex difference of this association needs further exploration.

The findings of subgroups suggested the sex difference of the relation between DM and gastrointestinal cancer might be variable according to pre-defined factors. First, we noted that publication year affected the sex difference on the risk of EC, GC, CRC, and CC. The possible reason for this could be that the diagnosis criteria and approach were different, which was associated with the risk of cancer at different sites. Further, the pooled RRR (female to male) was increased for EC and GC in Eastern countries, while this difference was reduced for CRC, CC, and HCC in Western countries. The reason for this could be that the prevalence of EC and GC was higher in Eastern countries than in Western countries and the prevalence of cancer between men and women was different. Third, the study design of included research might have affected the relation between DM and gastrointestinal cancer due to uncontrolled biases in studies with different designs. Fourth, sample size affected the sex differences on the risk of CC because the statistical power was enough to detect small differences. Fifth, different mean age affected the incidence of gastrointestinal cancer because the prevalence of EC, GC, and HCC was higher in younger individuals, while the incidence of CRC and CC was more common in older individuals. Sixth, the type of DM might affect the possible incidence of gastrointestinal cancer because patients with Type 1 DM were younger than those with Type 2 DM. Seventh, studies with longer follow-up and higher proportion of cancers than studies with shorter follow-up contributed higher weight to pooled results and more easily detected small sex differences. Finally, the confounders, whether adjusted or not, were affected by the sex difference relation between DM and gastrointestinal cancer. The possible reasons are presented as follows: (1) Patients with Type 2 DM always had higher BMIs, which might affect the incidence of cancer. This factor should adjust because individuals with higher BMIs more easily suffer DM, and participants with prediabetes were associated with the risk of cancer [83]. (2) Smoking is relatively infrequent in women in some regions of the world as compared with men; this factor was not adjusted in most included studies [21–26, 45–48, 52, 54, 55, 57–59, 61, 62, 64, 65, 67]. (3) Heavy alcohol intake was associated with higher risk of EC, GC, CRC, HCC, and PC, which might bias the relation between DM and gastrointestinal cancer. Further, the intake dose was different between men and women [84]. (4) PA may be protective against cancer risk, which might be because PA induces a decrease in circulating sex hormones and lower BMI [85, 86].

Four strengths of our study should be highlighted. First, only cohort studies were included, which should eliminate uncontrolled bias. Second, the large sample size allowed us to quantitatively assess the sex difference in the association of DM with the risk of gastrointestinal cancer, and thus, our findings are potentially more robust than are those of any individual study. Third, the consistency in the findings of this study and the lack of significant publication bias also support the robustness of the study findings. Finally, the study provided evidence supporting the sex difference of DM and gastrointestinal cancer risk in patients with specific characteristics.

The limitations of our study are as follows: (1) the adjusted models are different across the included studies, and these factors might play an important role in the development of gastrointestinal cancer; (2) inconsistencies among included studies in DM types, assessment of DM, and DM duration were identified; (3) the effect modification of gender on the associations of DM with gastrointestinal cancer were neglected due to data were not available in all of included studies; (4) stratified analyses based on large numbers of factors might induce multiple comparisons; and (5) the individual data were not available, and the findings of this study were based on pooled data, which restricted us from performing a more detailed relevant analysis and obtaining more comprehensive results.

## Conclusions

In summary, the findings of this meta-analysis suggested that women with DM was associated with an increased risk of GC, and reduced risk of HCC and CC as compared with men with DM. Further, a sex difference might exist for EC, and CRC. The true associations of DM with the risk of gastrointestinal cancer between men and women might be variable in the study of individuals with specific characteristics. Future studies should focus on specific populations and compare the association between DM and gastrointestinal cancer risk in groups of participants categorized by potential confounders.

## Additional files

Additional file 1: Newcastle–Ottawa scale for quality assessment of the included studies. (DOC 99 kb)

Additional file 2: STATA program for calculate the ratio of relative risk. (DOC 37 kb)

Additional file 3: Effect estimate of relationship between diabetes mellitus and gastrointestinal cancer in each included studies. (DOC 174 kb)

Additional file 4: The summary results for the relationship between diabetes mellitus and gastrointestinal cancer. (DOC 45 kb)

Additional file 5: The details of sensitivity analyses for gastrointestinal cancer. (DOC 254 kb)

Additional file 6: The findings of meta-regression for gastrointestinal cancer based on publication year, sample size, mean age, percentage of smoker, and follow-up duration. (DOC 36 kb)

Additional file 7: Subgroup analyses for the relationship between diabetes mellitus and gastrointestinal cancer in women compared with men. (DOC 643 kb)

Additional file 8: Publication biases for gastrointestinal cancer. (DOC 129 kb)

## Abbreviations

CC: Colon cancer
CI: Confidence interval
CRC: Colorectal cancer
DM: Diabetes mellitus
EC: Esophageal cancer
GC: Gastric cancer
HCC: Hepatocellular carcinoma
HR: Hazard ratio
IDF: International Diabetes Federation
OR: Odds ratio
PC: Pancreatic cancer
RC: Rectal cancer
RR: Relative risk
RRR: Ratio of relative risk
SIR: Standard incidence ratio
SMR: Standard mortality ratio
