# Supplementary material for: Diabetes mellitus and the risk of gastrointestinal cancer in women compared with men: a meta-analysis of cohort studies
Source: BMC Cancer. 2018 Apr 16;18:422. doi: 10.1186/s12885-018-4351-4 (PMC5902961; doi:10.1186/s12885-018-4351-4)
Supplement: Supplementary file 1 — Newcastle–Ottawa scale for quality assessment of the included studies. (DOC 99 kb) [file 12885_2018_4351_MOESM1_ESM.doc]

Table S1. Quality scores of prospective cohort studies using Newcastle-Ottawa Scale.

| Study | Selection | | | | Comparability | Outcome | | | NOS |
| --- | --- | --- | --- | --- | --- | --- | --- | --- | --- |
|  | Representativeness of the exposed cohort | Selection of the non exposed cohort | Ascertainment  of DM | Demonstration that outcomes was not present at start of study | Comparability on the basis of the design or analysis | Assessment of outcome | Adequate follow-up duration | Adequate follow-up rate | Overall score |
| Verona Diabetes  Study 2003 [38] | 1 | 0 | 1 | 1 | 1 | 1 | 1 | 1 | 7 |
| Veneto Region 2014 [26] | 1 | 0 | 1 | 1 | 1 | 1 | 0 | 1 | 6 |
| Uppsala Health Care Region 1991 [22] | 1 | 1 | 1 | 1 | 2 | 1 | 1 | 1 | 9 |
| Takayama Study cohort 2013 [39] | 1 | 1 | 1 | 1 | 2 | 1 | 1 | 1 | 9 |
| The Singapore  Chinese Health Study 2006/2013 [40,41] | 1 | 1 | 1 | 1 | 2 | 1 | 1 | 1 | 9 |
| SMHS and SWHS 2013/2015 [42,43] | 1 | 1 | 1 | 1 | 2 | 1 | 0 | 1 | 8 |
| Ragozzino 1982 [44] | 1 | 1 | 1 | 1 | 2 | 1 | 0 | 1 | 8 |
| Limburg 2006 [45] | 1 | 0 | 1 | 1 | 1 | 1 | 1 | 1 | 7 |
| PHARMO Database 2017 [46] | 1 | 1 | 1 | 1 | 2 | 1 | 1 | 1 | 9 |
| Gini 2016 [47] | 1 | 0 | 1 | 1 | 1 | 1 | 0 | 1 | 6 |
| NIH-AARP Diet and Health  Study 2011 [48] | 1 | 1 | 1 | 1 | 2 | 1 | 0 | 1 | 8 |
| Korean Cancer Prevention Study 2005 [49] | 1 | 1 | 1 | 1 | 2 | 1 | 1 | 1 | 9 |
| NHANESI 1995 [50] | 1 | 1 | 1 | 1 | 2 | 1 | 1 | 1 | 9 |
| The Cardiovascular Health Study 1999 [51] | 1 | 1 | 1 | 1 | 2 | 1 | 0 | 1 | 8 |
| Fujino 2001 [52] | 1 | 1 | 1 | 1 | 2 | 1 | 1 | 1 | 9 |
| Clalit Health Care Services 2013 [53] | 1 | 0 | 1 | 1 | 1 | 1 | 1 | 1 | 7 |
| Clalit Health Services 2016 [54] | 1 | 1 | 1 | 1 | 2 | 1 | 1 | 1 | 9 |
| Danish Central Hospital Discharge Register 1997 [25] | 1 | 0 | 1 | 1 | 1 | 1 | 0 | 1 | 6 |
| Cancer Prevention Study 1998 [55] | 1 | 1 | 1 | 1 | 2 | 1 | 1 | 1 | 9 |
| D2C cohort 2011 [56] | 1 | 0 | 1 | 1 | 1 | 1 | 0 | 1 | 6 |
| Diabetes Registry Tyrol 2014 [57] | 1 | 0 | 1 | 1 | 1 | 1 | 0 | 1 | 6 |
| Koskinen 1998 [58] | 1 | 0 | 1 | 1 | 1 | 1 | 0 | 1 | 6 |
| EPIC-Norfolk Study 2004 [59] | 1 | 1 | 1 | 1 | 2 | 1 | 0 | 1 | 8 |
| Xu 2015 [60] | 1 | 0 | 1 | 1 | 1 | 1 | 0 | 1 | 6 |
| Newfoundland and Labrador 2013 [61] | 1 | 1 | 1 | 1 | 2 | 1 | 1 | 1 | 9 |
| Netherlands Cohort Study 2016 [62] | 1 | 1 | 1 | 1 | 2 | 1 | 1 | 1 | 9 |
| Maccabi  Healthcare Services 2010 [63] | 1 | 1 | 1 | 1 | 2 | 1 | 0 | 1 | 8 |
| National Health Screening Service 2001 [21] | 1 | 1 | 1 | 1 | 2 | 1 | 1 | 1 | 9 |
| Nationwide Cohort Study in Sweden 1995 [64] | 1 | 0 | 1 | 1 | 1 | 1 | 0 | 1 | 6 |
| The Multiethnic Cohort 2010 [65] | 1 | 1 | 1 | 1 | 2 | 1 | 1 | 1 | 9 |
| Wang 2015 [23] | 1 | 0 | 1 | 1 | 1 | 1 | 0 | 1 | 6 |
| Zhang 2012 [66,67] | 1 | 0 | 1 | 1 | 1 | 1 | 0 | 1 | 6 |
| Japan Public Health Center- Based Prospective Study 2006 [68,69] | 1 | 1 | 1 | 1 | 2 | 1 | 1 | 1 | 9 |
| Cancer Prevention Study II 2004 [19,20] | 1 | 1 | 1 | 1 | 2 | 1 | 1 | 1 | 9 |
| Japan Collaborative Cohort Study 2006 [70-71] | 1 | 1 | 1 | 1 | 2 | 1 | 0 | 1 | 8 |
| National Health Insurance Program 2014 [24] | 1 | 0 | 1 | 1 | 1 | 1 | 1 | 1 | 7 |
| Zhou 2010 [72] | 1 | 1 | 1 | 1 | 2 | 1 | 0 | 1 | 8 |
| EPOCH-JAPAN 2017 [73] | 1 | 1 | 1 | 1 | 2 | 1 | 1 | 1 | 9 |
