# Supplementary material for: Diabetes mellitus and the risk of gastrointestinal cancer in women compared with men: a meta-analysis of cohort studies
Source: BMC Cancer. 2018 Apr 16;18:422. doi: 10.1186/s12885-018-4351-4 (PMC5902961; doi:10.1186/s12885-018-4351-4)

**The details of STATA program for calculate ratio of RRs:**

gen logrr1=log(rr1)

gen logrr2=log(rr2)

gen logrrl1=log(rrl1)

gen logrru1=log(rru1)

gen logrrl2=log(rrl2)

gen logrru2=log(rru2)

gen selogrr1=(log(rru1)-log(rrl1))/(2*1.96)

gen selogrr2=(log(rru2)-log(rrl2))/(2*1.96)

gen logrr= logrr1- logrr2

gen logrrl= logrr-1.96*( selogrr1^2+ selogrr2^2)^(0.5)

gen logrru= logrr+1.96*( selogrr1^2+ selogrr2^2)^(0.5)

sort logrr

metan logrr logrrl logrru, eform random xlab(0.3, 0.5, 1.0, 2.0) effect(rrr) lcols(study) texts(110)

The data presented layout are presented as follows (Esophagus cancer data):


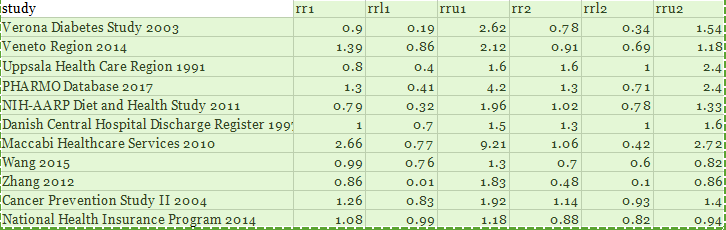

Supplement: Supplementary file 2 — STATA program for calculate the ratio of relative risk. (DOC 37 kb) [file 12885_2018_4351_MOESM2_ESM.doc]
