# Supplementary material for: Diabetes mellitus and the risk of gastrointestinal cancer in women compared with men: a meta-analysis of cohort studies
Source: BMC Cancer. 2018 Apr 16;18:422. doi: 10.1186/s12885-018-4351-4 (PMC5902961; doi:10.1186/s12885-018-4351-4)
Supplement: Supplementary file 3 — Effect estimate of relationship between diabetes mellitus and gastrointestinal cancer in each included studies. (DOC 174 kb) [file 12885_2018_4351_MOESM3_ESM.doc]

Table S1. Effect estimate of relationship between DM and gastrointestinal cancer in each included studies

| Study | Gender | Esophagus cancer | Gastric cancer | Colorectal cancer | Colon cancer | Rectal cancer | Hepatocellular carcinoma | Pancreatic cancer |
| --- | --- | --- | --- | --- | --- | --- | --- | --- |
| Verona Diabetes  Study 2003 [38] | Men | 0.78 (0.34-1.54) | 1.16 (0.77-1.68) | 1.18 (0.85-1.60) | - | - | 1.80 (1.29-2.46) | 0.90 (0.46-1.57) |
|  | Women | 0.9 (0.19-2.62) | 1.15 (0.70-1.77) | 0.78 (0.50-1.16) | - | - | 1.97 (1.26-2.91) | 1.78 (1.13-2.67) |
| Veneto Region 2014 [26] | Men | 0.91 (0.69-1.18) | 1.14 (0.96-1.33) | 1.22 (1.09-1.36) | - | - | 2.40 (2.19-2.62) | 2.16 (1.93-2.40) |
|  | Women | 1.39 (0.86-2.12) | 1.25 (0.99-1.56) | 1.13 (0.98-1.31) | - | - | 1.86 (1.56-2.19) | 1.95 (1.71-2.22) |
| Uppsala Health Care Region 1991 [22] | Men | 1.60 (1.00-2.40) | 0.80 (0.70-1.00) | - | 1.20 (0.90-1.40) | 1.30 (1.10-1.70) | 1.80 (1.40-2.30) | 1.40 (1.10-1.80) |
|  | Women | 0.80 (0.40-1.60) | 0.90 (0.70-1.20) | - | 1.00 (0.80-1.20) | 0.90 (0.70-1.20) | 1.30 (1.10-1.60) | 1.50 (1.20-1.80) |
| Takayama Study cohort 2013 [39] | Men | - | 1.20 (0.85-1.69) | - | 0.82 (0.48-1.39) | 1.27 (0.70-2.30) | 2.18 (1.27-3.74) | 1.63 (0.81-3.29) |
|  | Women | - | 2.15 (1.30-3.54) | - | 0.95 (0.47-1.93) | 1.81 (0.73-4.49) | 0.65 (0.16-2.69) | 1.53 (0.55-4.26) |
| The Singapore  Chinese Health Study 2006/2013 [40,41] | Men | - | - | 1.50 (1.20-2.10) | - | - | 2.11 (1.58-2.81) | - |
|  | Women | - | - | 1.40 (1.00-1.90) | - | - | 2.14 (1.41-3.25) | - |
| SMHS and SWHS 2013/2015 [42,43] | Men | - | 0.83 (0.59-1.16) | - | - | - | 1.63 (1.06-2.51) | - |
|  | Women | - | 0.92 (0.68-1.25) | - | - | - | 1.64 (1.03-2.61) | - |
| Ragozzino 1982 [44] | Men | - | 0.90 (0.20-2.70) | 1.40 (0.70-2.50) | - | - | - | 3.80 (1.20-9.00) |
|  | Women | - | 1.00 (0.10-3.40) | 1.10 (0.50-2.10) | - | - | - | 4.40 (1.20-11.40) |
| Limburg 2006 [45] | Men | - | - | 1.67 (1.16-2.33) | - | - | - | - |
|  | Women | - | - | 1.03 (0.60-1.66) | - | - | - | - |
| PHARMO Database 2017 [46] | Men | 1.30 (0.71-2.40) | 0.77 (0.47-1.30) | - | 1.60 (1.20-2.20) | 0.91 (0.59-1.40) | - | 5.00 (2.80-8.80) |
|  | Women | 1.30 (0.41-4.20) | 1.40 (0.55-3.40) | - | 1.10 (0.82-1.50) | 0.83 (0.48-1.50) | - | 4.50 (2.40-8.30) |
| Gini 2016 [47] | Men | - | - | 1.44 (1.25-1.65) | - | - | 2.25 (1.81-2.76) | 2.17 (1.60-2.87) |
|  | Women | - | - | 1.24 (1.02-1.50) | - | - | 1.83 (1.14-2.77) | 3.18 (2.46-4.04) |
| NIH-AARP Diet and Health  Study 2011 [48] | Men | 1.02 (0.78-1.33) | 1.32 (0.64-2.73) | - | - | - | - | - |
|  | Women | 0.79 (0.32-1.96) | 1.38 (0.82-2.30) | - | - | - | - | - |
| Korean Cancer Prevention Study 2005 [49] | Men | - | 1.16 (1.04-1.28) | 1.28 (1.06-1.55) | - | - | 1.59 (1.45-1.74) | 1.71 (1.42-2.06) |
|  | Women | - | 1.09 (0.88-1.36) | 1.11 (0.81-1.51) | - | - | 1.28 (1.00-1.66) | 1.71 (1.25-2.34) |
| NHANESI 1995 [50] | Men | - | - | 1.43 (0.61-3.31) | - | - | - | - |
|  | Women | - | - | 1.40 (0.64-3.10) | - | - | - | - |
| The Cardiovascular Health Study 1999 [51] | Men | - | - | 1.60 (0.80-3.10) | - | - | - | - |
|  | Women | - | - | 1.10 (0.50-2.60) | - | - | - | - |
| Fujino 2001 [52] | Men | - | - | - | - | - | 1.56 (0.66-3.67) | - |
|  | Women | - | - | - | - | - | 1.75 (0.32-9.60) | - |
| Clalit Health Care Services 2013 [53] | Men | - | - | 1.05 (0.76-1.34) | - | - | - | 3.27 (1.49-5.05) |
|  | Women | - | - | 1.30 (0.98-1.62) | - | - | - | 2.87 (1.25-4.50) |
| Clalit Health Services 2016 [54] | Men | - | 1.69 (1.50-1.90) | 1.45 (1.37-1.55) | - | - | 3.00 (2.49-3.61) | 3.25 (2.85-3.69) |
|  | Women | - | 1.90 (1.65-2.19) | 1.48 (1.39-1.57) | - | - | 3.50 (2.74-4.48) | 3.10 (2.72-3.52) |
| Danish Central Hospital  Discharge Register 1997 [25] | Men | 1.30 (1.00-1.60) | 1.20 (1.10-1.30) | - | 1.30 (1.10-1.40) | 1.10 (0.90-1.20) | 4.00 (3.50-4.60) | 1.70 (1.50-2.00) |
|  | Women | 1.00 (0.70-1.50) | 1.10 (1.00-1.40) | - | 1.10 (1.00-1.20) | 1.00 (0.90-1.20) | 2.10 (1.60-2.70) | 1.60 (1.40-1.90) |
| Cancer Prevention Study 1998 [55] | Men | - | - | 1.30 (1.03-1.65) | - | - | - | - |
|  | Women | - | - | 1.16 (0.87-1.53) | - | - | - | - |
| D2C cohort 2011 [56] | Men | - | - | 1.00 (0.77-1.23) | - | - | 1.88 (1.02-3.15) | 1.27 (0.68-2.15) |
|  | Women | - | - | 0.97 (0.73-1.28) | - | - | 2.08 (0.78-4.46) | 1.63 (0.93-2.63) |
| Diabetes Registry Tyrol 2014 [57] | Men | - | 0.55 (0.24-1.08) | 1.11 (0.81-1.49) | - | - | 2.71 (1.65-4.18) | 1.87 (1.11-2.96) |
|  | Women | - | 1.07 (0.51-1.97) | 0.94 (0.62-1.36) | - | - | 2.40 (0.96-4.94) | 1.78 (1.02-2.89) |
| Koskinen 1998 [58] | Men | - | 1.40 (1.11-1.77) | - | 1.26 (0.93-1.71) | - | - | - |
|  | Women | - | 1.39 (1.09-1.78) | - | 1.30 (1.02-1.67) | - | - | - |
| EPIC-Norfolk Study 2004 [59] | Men | - | - | 6.02 (1.47-24.65) | - | - | - | - |
|  | Women | - | - | 1.38 (0.16-11.76) | - | - | - | - |
| Xu 2015 [60] | Men | - | 1.17 (0.90-1.43) | - | 1.97 (1.49-2.46) | 1.72 (1.23-2.21) | 1.20 (0.87-1.53) | 1.28 (0.77-1.80) |
|  | Women | - | 1.26 (0.91-1.61) | - | 1.67 (1.25-2.08) | 1.28 (0.82-1.73) | 1.09 (0.65-1.53) | 0.96 (0.51-1.40) |
| Newfoundland and Labrador 2013 [61] | Men | - | - | 1.38 (1.19-1.60) | 1.49 (1.24-1.78) | 1.19 (0.93-1.53) | - | - |
|  | Women | - | - | 1.52 (1.27-1.80) | 1.47 (1.20-1.80) | 1.56 (1.10-2.22) | - | - |
| Netherlands Cohort Study 2016 [62] | Men | - | - | 0.95 (0.75-1.20) | 0.77 (0.49-1.21) | 0.50 (0.21-1.22) | - | - |
|  | Women | - | - | 1.08 (0.85-1.37) | 0.75 (0.44-1.27) | 1.16 (0.54-2.48) | - | - |
| Maccabi  Healthcare Services 2010 [63] | Men | 1.06 (0.42-2.72) | 1.44 (0.98-2.11) | - | 1.14 (0.91-1.44) | 1.10 (0.74-1.61) | 1.83 (0.86-3.89) | 1.47 (0.90-2.41) |
|  | Women | 2.66 (0.77-9.21) | 0.99 (0.55-1.80) | - | 1.52 (1.19-1.95) | 1.08 (0.69-1.69) | 2.42 (1.00-5.84) | 1.89 (1.16-3.07) |
| National Health Screening Service 2001 [21] | Men | - | - | 0.66 (0.35-1.24) | 0.88 (0.39-1.99) | - | - | - |
|  | Women | - | - | 1.55 (1.04-2.31) | 1.12 (0.59-2.14) | - | - | - |
| Nationwide Cohort Study in Sweden 1995 [64] | Men | - | - | - | - | - | - | 1.88 (1.67-2.10) |
|  | Women | - | - | - | - | - | - | 1.97 (1.77-2.19) |
| The Multiethnic Cohort 2010 [65] | Men | - | - | 1.12 (0.99-1.26) | - | - | - | - |
|  | Women | - | - | 1.28 (1.12-1.46) | - | - | - | - |
| Wang 2015 [23] | Men | 0.70 (0.60-0.82) | 0.82 (0.75-0.90) | - | 1.47 (1.29-1.67) | 1.25 (1.09-1.43) | 1.26 (1.16-1.36) | 2.81 (2.50-3.16) |
|  | Women | 0.99 (0.76-1.30) | 1.16 (1.03-1.30) | - | 1.33 (1.15-1.54) | 1.29 (1.10-1.51) | 1.53 (1.35-1.73) | 3.62 (3.20-4.09) |
| Zhang 2012 [66,67] | Men | 0.48 (0.10-0.86) | 1.22 (0.74-1.70) | 1.82 (1.23-2.41) | 1.90 (1.14-2.66) | 1.72 (0.79-2.66) | 1.54 (1.01-2.07) | 2.97 (1.73-4.22) |
|  | Women | 0.86 (0.01-1.83) | 1.55 (0.79-2.32) | 1.36 (0.85-1.88) | 1.69 (0.95-2.43) | 0.91 (0.24-1.58) | 1.46 (0.74-2.17) | 2.69 (1.45-3.93) |
| Japan Public Health Center- Based Prospective Study 2006 [68,69] | Men | - | 1.23 (0.98-1.54) | - | 1.36 (1.00-1.85) | 0.80 (0.47-1.36) | 2.24 (1.64-3.04) | 1.85 (1.07-3.20) |
|  | Women | - | 1.61 (1.02-2.54) | - | 0.83 (0.42-1.61) | 1.65 (0.80-3.39) | 1.94 (1.00-3.73) | 1.33 (0.53-3.31) |
| Cancer Prevention Study II 2004 [19,20] | Men | 1.14 (0.93-1.40) | 0.99 (0.79-1.22) | - | 1.15 (1.03-1.29) | 1.01 (0.75-1.36) | 2.26 (1.89-2.70) | 1.40 (1.23-1.59) |
|  | Women | 1.26 (0.83-1.92) | 1.24 (0.95-1.63) | - | 1.18 (1.04-1.33) | 0.79 (0.54-1.16) | 1.40 (1.05-1.86) | 1.31 (1.14-1.51) |
| Japan Collaborative Cohort Study 2006 [70-71] | Men | - | 0.67 (0.46-0.99) | 1.30 (0.80-1.99) | 1.33 (0.79-2.23) | 0.95 (0.48-1.88) | 2.30 (1.47-3.59) | 1.97 (0.93-4.19) |
|  | Women | - | 0.49 (0.23-1.04) | 1.70 (1.00-3.00) | 1.00 (0.46-2.15) | 2.54 (0.89-7.25) | 2.70 (1.20-6.05) | 1.42 (0.61-3.29) |
| National Health Insurance Program 2014 [24] | Men | 0.88 (0.82-0.94) | 0.96 (0.92-1.00) | 1.19 (1.15-1.22) | - | - | 1.61 (1.57-1.64) | 1.62 (1.53-1.72) |
|  | Women | 1.08 (0.99-1.18) | 1.11 (1.05-1.17) | 1.16 (1.13-1.20) | - | - | 1.55 (1.51-1.60) | 1.44 (1.34-1.55) |
| Zhou 2010 [72] | Men | - | - | - | - | - | 5.16 (2.56-10.41) | 1.67 (0.94-2.97) |
|  | Women | - | - | - | - | - | 6.37 (2.18-18.62) | 2.13 (1.09-4.16) |
| EPOCH-JAPAN 2017 [73] | Men | - | - | - | - | - | - | 1.84 (0.88-3.84) |
|  | Women | - | - | - | - | - | - | 1.02 (0.28-3.72) |
