# Supplementary material for: Diabetes mellitus and the risk of gastrointestinal cancer in women compared with men: a meta-analysis of cohort studies
Source: BMC Cancer. 2018 Apr 16;18:422. doi: 10.1186/s12885-018-4351-4 (PMC5902961; doi:10.1186/s12885-018-4351-4)
Supplement: Supplementary file 4 — The summary results for the relationship between diabetes mellitus and gastrointestinal cancer. (DOC 45 kb) [file 12885_2018_4351_MOESM4_ESM.doc]

Table S1. The summary results for the relationship between DM and gastrointestinal cancer

| Outcomes | Effect estimate | RR and 95% CI for women | P value | Heterogeneity (%) | P value for heterogeneity | RR and 95% CI for men | P value | Heterogeneity (%) | P value for heterogeneity |
| --- | --- | --- | --- | --- | --- | --- | --- | --- | --- |
| Esophagus cancer | SIR/SMR | 1.08 (0.99-1.17) | 0.074 | 0.0 | 0.869 | 0.89 (0.73-1.07) | 0.208 | 75.2 | 0.001 |
|  | RR/OR/HR | 1.14 (0.84-1.56) | 0.397 | 0.0 | 0.449 | 1.15 (1.00-1.33) | 0.058 | 0.0 | 0.532 |
| Gastric cancer | SIR/SMR | 1.14 (1.09-1.19) | <0.001 | 0.0 | 0.640 | 1.07 (0.95-1.21) | 0.284 | 81.5 | <0.001 |
|  | RR/OR/HR | 1.22 (0.96-1.54) | 0.099 | 79.3 | <0.001 | 1.07 (0.88-1.28) | 0.503 | 84.9 | <0.001 |
| Colorectal cancer | SIR/SMR | 1.15 (1.11-1.20) | <0.001 | 3.0 | 0.409 | 1.25 (1.14-1.37) | <0.001 | 59.6 | 0.011 |
|  | RR/OR/HR | 1.36 (1.25-1.47) | <0.001 | 22.9 | 0.212 | 1.28 (1.14-1.43) | <0.001 | 64.2 | 0.001 |
| Colon cancer | SIR/SMR | 1.33 (1.13-1.57) | 0.001 | 71.9 | 0.007 | 1.50 (1.28-1.75) | <0.001 | 64.4 | 0.024 |
|  | RR/OR/HR | 1.17 (1.03-1.33) | 0.019 | 42.6 | 0.074 | 1.22 (1.08-1.38) | 0.001 | 45.5 | 0.057 |
| Rectal cancer | SIR/SMR | 1.15 (0.96-1.37) | 0.133 | 50.7 | 0.107 | 1.31 (1.09-1.58) | 0.005 | 65.0 | 0.036 |
|  | RR/OR/HR | 1.13 (0.90-1.42) | 0.289 | 44.9 | 0.069 | 1.10 (0.97-1.25) | 0.127 | 6.3 | 0.383 |
| Hepatocellular carcinoma | SIR/SMR | 1.65 (1.51-1.82) | <0.001 | 41.1 | 0.083 | 1.94 (1.56-2.41) | <0.001 | 97.0 | <0.001 |
|  | RR/OR/HR | 1.91 (1.42-2.56) | <0.001 | 80.0 | <0.001 | 2.14 (1.78-2.57) | <0.001 | 79.1 | <0.001 |
| Pancreatic cancer | SIR/SMR | 2.01 (1.60-2.51) | <0.001 | 94.3 | <0.001 | 1.92 (1.64-2.25) | <0.001 | 88.6 | <0.001 |
|  | RR/OR/HR | 1.94 (1.42-2.64) | <0.001 | 88.9 | <0.001 | 1.99 (1.50-2.64) | <0.001 | 89.8 | <0.001 |
