# Supplementary material for: Diabetes mellitus and the risk of gastrointestinal cancer in women compared with men: a meta-analysis of cohort studies
Source: BMC Cancer. 2018 Apr 16;18:422. doi: 10.1186/s12885-018-4351-4 (PMC5902961; doi:10.1186/s12885-018-4351-4)
Supplement: Supplementary file 5 — The details of sensitivity analyses for gastrointestinal cancer. (DOC 254 kb) [file 12885_2018_4351_MOESM5_ESM.doc]

Table S1. The details of sensitivity analysis for esophagus cancer based on SIR/SMR and RR/OR/HR

| **Effect estimate** | **Excluding study** | **RRR and 95% CI** | **P value** | **Heterogeneity（%）** | **P value for heterogeneity** |
| --- | --- | --- | --- | --- | --- |
| SIR/SMR | Verona Diabetes Study 2003 | 1.22 (1.01-1.47) | 0.035 | 30.1 | 0.221 |
|  | Veneto Region 2014 | 1.19 (1.00-1.42) | 0.047 | 20.5 | 0.284 |
|  | Danish Central Hospital Discharge Register 1997 | 1.26 (1.13-1.39) | <0.001 | 0.0 | 0.856 |
|  | Wang 2015 | 1.17 (0.95-1.43) | 0.135 | 17.1 | 0.305 |
|  | Zhang 2012 | 1.22 (1.02-1.46) | 0.033 | 29.3 | 0.226 |
|  | National Health Insurance Program 2014 | 1.20 (0.88-1.65) | 0.258 | 30.2 | 0.221 |
| RR/OR/HR | Uppsala Health Care Region 1991 | 1.09 (0.74-1.60) | 0.675 | 0.0 | 0.653 |
|  | PHARMO Database 2017 | 0.91 (0.55-1.50) | 0.708 | 32.4 | 0.218 |
|  | NIH-AARP Diet and Health Study 2011 | 0.95 (0.56-1.61) | 0.860 | 29.4 | 0.236 |
|  | Maccabi Healthcare Services 2010 | 0.90 (0.63-1.28) | 0.547 | 0.0 | 0.417 |
|  | Cancer Prevention Study II 2004 | 0.79 (0.45-1.41) | 0.430 | 12.8 | 0.329 |

Table S2. The details of sensitivity analysis for gastric cancer based on SIR/SMR and RR/OR/HR

| **Effect estimate** | **Excluding study** | **RRR and 95% CI** | **P value** | **Heterogeneity（%）** | **P value for heterogeneity** |
| --- | --- | --- | --- | --- | --- |
| SIR/SMR | Verona Diabetes Study 2003 | 1.15 (1.02-1.29) | 0.024 | 49.2 | 0.055 |
|  | Veneto Region 2014 | 1.14 (1.01-1.30) | 0.039 | 49.6 | 0.053 |
|  | Danish Central Hospital Discharge Register 1997 | 1.19 (1.09-1.31) | <0.001 | 21.5 | 0.259 |
|  | Diabetes Registry Tyrol 2014 | 1.13 (1.01-1.27) | 0.029 | 46.4 | 0.071 |
|  | Koskinen 1998 | 1.16 (1.02-1.30) | 0.019 | 46.9 | 0.068 |
|  | Xu 2015 | 1.15 (1.01-1.30) | 0.032 | 49.6 | 0.053 |
|  | Wang 2015 | 1.13 (1.06-1.20) | <0.001 | 0.0 | 0.518 |
|  | Zhang 2012 | 1.14 (1.01-1.28) | 0.035 | 50.0 | 0.051 |
|  | National Health Insurance Program 2014 | 1.13 (0.95-1.33) | 0.167 | 49.9 | 0.052 |
| RR/OR/HR | Uppsala Health Care Region 1991 | 1.10 (0.98-1.24) | 0.116 | 0.0 | 0.565 |
|  | Takayama Study cohort 2013 | 1.08 (0.97-1.21) | 0.161 | 0.0 | 0.804 |
|  | SMHS and SWHS 2013/2015 | 1.10 (0.98-1.23) | 0.097 | 0.0 | 0.563 |
|  | Ragozzino 1982 | 1.10 (0.99-1.23) | 0.086 | 0.0 | 0.563 |
|  | PHARMO Database 2017 | 1.10 (0.98-1.22) | 0.107 | 0.0 | 0.650 |
|  | NIH-AARP Diet and Health Study 2011 | 1.10 (0.99-1.23) | 0.085 | 0.0 | 0.564 |
|  | Korean Cancer Prevention Study 2005 | 1.15 (1.02-1.30) | 0.028 | 0.0 | 0.767 |
|  | Clalit Health Services 2016 | 1.09 (0.95-1.25) | 0.224 | 0.0 | 0.570 |
|  | Maccabi Healthcare Services 2010 | 1.12 (1.00-1.25) | 0.057 | 0.0 | 0.733 |
|  | Japan Public Health Center- Based Prospective Study 2006 | 1.09 (0.98-1.22) | 0.126 | 0.0 | 0.608 |
|  | Cancer Prevention Study II 2004 | 1.09 (0.97-1.22) | 0.166 | 0.0 | 0.620 |
|  | Japan Collaborative Cohort Study 2006 | 1.11 (0.99-1.24) | 0.067 | 0.0 | 0.652 |

Table S3. The details of sensitivity analysis for colorectal cancer based on SIR/SMR and RR/OR/HR

| **Effect estimate** | **Excluding study** | **RRR and 95% CI** | **P value** | **Heterogeneity（%）** | **P value for heterogeneity** |
| --- | --- | --- | --- | --- | --- |
| SIR/SMR | Verona Diabetes Study 2003 | 0.97 (0.93-1.01) | 0.101 | 0.0 | 0.521 |
|  | Veneto Region 2014 | 0.94 (0.86-1.03) | 0.186 | 12.0 | 0.337 |
|  | Limburg 2006 | 0.97 (0.93-1.01) | 0.098 | 0.0 | 0.525 |
|  | Gini 2016 | 0.96 (0.91-1.02) | 0.188 | 3.4 | 0.404 |
|  | Clalit Health Care Services 2013 | 0.96 (0.93-1.00) | 0.061 | 0.0 | 0.484 |
|  | D2C cohort 2011 | 0.94 (0.87-1.02) | 0.133 | 14.2 | 0.319 |
|  | Diabetes Registry Tyrol 2014 | 0.95 (0.88-1.02) | 0.153 | 11.3 | 0.342 |
|  | Zhang 2012 | 0.96 (0.91-1.01) | 0.139 | 3.1 | 0.406 |
|  | National Health Insurance Program 2014 | 0.90 (0.80-1.01) | 0.065 | 0.0 | 0.492 |
| RR/OR/HR | The Singapore Chinese Health Study 2006/2013 | 1.04 (0.97-1.12) | 0.218 | 0.0 | 0.480 |
|  | Ragozzino 1982 | 1.04 (0.97-1.12) | 0.229 | 0.0 | 0.487 |
|  | Korean Cancer Prevention Study 2005 | 1.05 (0.98-1.12) | 0.184 | 0.0 | 0.546 |
|  | NHANESI 1995 | 1.04 (0.97-1.11) | 0.242 | 0.0 | 0.459 |
|  | The Cardiovascular Health Study 1999 | 1.04 (0.97-1.12) | 0.226 | 0.0 | 0.509 |
|  | Clalit Health Services 2016 | 1.07 (0.96-1.20) | 0.200 | 0.0 | 0.502 |
|  | Cancer Prevention Study 1998 | 1.05 (0.98-1.12) | 0.194 | 0.0 | 0.519 |
|  | EPIC-Norfolk Study 2004 | 1.04 (0.97-1.11) | 0.232 | 0.0 | 0.577 |
|  | Newfoundland and Labrador 2013 | 1.04 (0.96-1.11) | 0.335 | 0.0 | 0.480 |
|  | Netherlands Cohort Study 2016 | 1.04 (0.97-1.11) | 0.301 | 0.0 | 0.482 |
|  | National Health Screening Service 2001 | 1.03 (0.97-1.11) | 0.334 | 0.0 | 0.857 |
|  | The Multiethnic Cohort 2010 | 1.02 (0.95-1.10) | 0.507 | 0.0 | 0.566 |
|  | Japan Collaborative Cohort Study 2006 | 1.04 (0.97-1.11) | 0.271 | 0.0 | 0.492 |

Table S4. The details of sensitivity analysis for colon cancer based on SIR/SMR and RR/OR/HR

| **Effect estimate** | **Excluding study** | **RRR and 95% CI** | **P value** | **Heterogeneity（%）** | **P value for heterogeneity** |
| --- | --- | --- | --- | --- | --- |
| SIR/SMR | Danish Central Hospital Discharge Register 1997 | 0.91 (0.78-1.06) | 0.231 | 0.0 | 0.907 |
|  | Koskinen 1998 | 0.87 (0.77-0.97) | 0.012 | 0.0 | 0.959 |
|  | Xu 2015 | 0.88 (0.79-0.99) | 0.027 | 0.0 | 0.808 |
|  | Wang 2015 | 0.87 (0.76-0.99) | 0.029 | 0.0 | 0.831 |
|  | Zhang 2012 | 0.88 (0.79-0.98) | 0.019 | 0.0 | 0.799 |
| RR/OR/HR | Uppsala Health Care Region 1991 | 1.00 (0.89-1.14) | 0.964 | 2.9 | 0.411 |
|  | Takayama Study cohort 2013 | 0.97 (0.85-1.10) | 0.606 | 14.8 | 0.310 |
|  | PHARMO Database 2017 | 1.00 (0.90-1.13) | 0.934 | 0.0 | 0.568 |
|  | Newfoundland and Labrador 2013 | 0.96 (0.83-1.12) | 0.620 | 16.0 | 0.300 |
|  | Netherlands Cohort Study 2016 | 0.97 (0.85-1.11) | 0.642 | 16.1 | 0.300 |
|  | Maccabi Healthcare Services 2010 | 0.94 (0.84-1.06) | 0.336 | 0.0 | 0.654 |
|  | National Health Screening Service 2001 | 0.97 (0.85-1.10) | 0.604 | 13.8 | 0.319 |
|  | Japan Public Health Center- Based Prospective Study 2006 | 0.99 (0.89-1.11) | 0.866 | 0.0 | 0.442 |
|  | Cancer Prevention Study II 2004 | 0.94 (0.80-1.11) | 0.467 | 11.2 | 0.342 |
|  | Japan Collaborative Cohort Study 2006 | 0.98 (0.86-1.11) | 0.705 | 13.2 | 0.325 |

Table S5. The details of sensitivity analysis for rectal cancer based on SIR/SMR and RR/OR/HR

| **Effect estimate** | **Excluding study** | **RRR and 95% CI** | **P value** | **Heterogeneity（%）** | **P value for heterogeneity** |
| --- | --- | --- | --- | --- | --- |
| SIR/SMR | Danish Central Hospital Discharge Register 1997 | 0.91 (0.69-1.21) | 0.508 | 24.6 | 0.265 |
|  | Xu 2015 | 0.96 (0.83-1.11) | 0.555 | 0.0 | 0.402 |
|  | Wang 2015 | 0.87 (0.72-1.05) | 0.136 | 0.0 | 0.509 |
|  | Zhang 2012 | 0.95 (0.82-1.09) | 0.430 | 0.0 | 0.407 |
| RR/OR/HR | Uppsala Health Care Region 1991 | 1.18 (0.90-1.55) | 0.226 | 16.5 | 0.300 |
|  | Takayama Study cohort 2013 | 1.08 (0.79-1.47) | 0.632 | 48.9 | 0.057 |
|  | PHARMO Database 2017 | 1.13 (0.82-1.56) | 0.464 | 50.4 | 0.049 |
|  | Newfoundland and Labrador 2013 | 1.05 (0.76-1.45) | 0.760 | 41.8 | 0.100 |
|  | Netherlands Cohort Study 2016 | 1.04 (0.78-1.38) | 0.805 | 41.8 | 0.100 |
|  | Maccabi Healthcare Services 2010 | 1.13 (0.81-1.58) | 0.481 | 50.6 | 0.048 |
|  | Japan Public Health Center- Based Prospective Study 2006 | 1.02 (0.77-1.35) | 0.897 | 38.7 | 0.121 |
|  | Cancer Prevention Study II 2004 | 1.17 (0.84-1.64) | 0.344 | 46.7 | 0.069 |
|  | Japan Collaborative Cohort Study 2006 | 1.03 (0.78-1.36) | 0.829 | 40.0 | 0.112 |

Table S6. The details of sensitivity analysis for hepatocellular carcinoma based on SIR/SMR and RR/OR/HR

| **Effect estimate** | **Excluding study** | **RRR and 95% CI** | **P value** | **Heterogeneity（%）** | **P value for heterogeneity** |
| --- | --- | --- | --- | --- | --- |
| SIR/SMR | Verona Diabetes Study 2003 | 0.88 (0.74-1.04) | 0.133 | 74.4 | <0.001 |
|  | Veneto Region 2014 | 0.91 (0.76-1.10) | 0.339 | 69.8 | 0.001 |
|  | Gini 2016 | 0.90 (0.75-1.06) | 0.213 | 74.2 | <0.001 |
|  | Danish Central Hospital Discharge Register 1997 | 0.97 (0.86-1.10) | 0.632 | 47.3 | 0.055 |
|  | D2C cohort 2011 | 0.89 (0.75-1.05) | 0.154 | 74.5 | <0.001 |
|  | Diabetes Registry Tyrol 2014 | 0.89 (0.75-1.05) | 0.173 | 74.5 | <0.001 |
|  | Xu 2015 | 0.89 (0.75-1.06) | 0.179 | 74.5 | <0.001 |
|  | Wang 2015 | 0.83 (0.69-1.00) | 0.045 | 62.1 | 0.007 |
|  | Zhang 2012 | 0.89 (0.75-1.05) | 0.167 | 74.6 | <0.001 |
|  | National Health Insurance Program 2014 | 0.87 (0.68-1.12) | 0.288 | 74.4 | <0.001 |
| RR/OR/HR | Uppsala Health Care Region 1991 | 0.88 (0.74-1.05) | 0.167 | 12.5 | 0.325 |
|  | Takayama Study cohort 2013 | 0.86 (0.74-1.00) | 0.046 | 5.9 | 0.388 |
|  | The Singapore Chinese Health Study 2006/2013 | 0.84 (0.71-1.00) | 0.052 | 16.7 | 0.285 |
|  | SMHS and SWHS 2013/2015 | 0.85 (0.72-1.01) | 0.060 | 18.1 | 0.271 |
|  | Korean Cancer Prevention Study 2005 | 0.87 (0.72-1.06) | 0.175 | 18.5 | 0.268 |
|  | Fujino 2001 | 0.86 (0.73-1.01) | 0.067 | 19.4 | 0.258 |
|  | Clalit Health Services 2016 | 0.79 (0.68-0.92) | 0.002 | 0.0 | 0.678 |
|  | Maccabi Healthcare Services 2010 | 0.85 (0.72-1.00) | 0.050 | 16.2 | 0.290 |
|  | Japan Public Health Center- Based Prospective Study 2006 | 0.86 (0.72-1.02) | 0.081 | 19.9 | 0.254 |
|  | Cancer Prevention Study II 2004 | 0.91 (0.78-1.05) | 0.203 | 0.0 | 0.591 |
|  | Japan Collaborative Cohort Study 2006 | 0.85 (0.72-1.00) | 0.051 | 16.8 | 0.284 |
|  | Zhou 2010 | 0.85 (0.72-1.01) | 0.057 | 17.8 | 0.274 |

Table S7. The details of sensitivity analysis for pancreatic cancer based on SIR/SMR and RR/OR/HR

| **Effect estimate** | **Excluding study** | **RRR and 95% CI** | **P value** | **Heterogeneity（%）** | **P value for heterogeneity** |
| --- | --- | --- | --- | --- | --- |
| SIR | Verona Diabetes Study 2003 | 1.02 (0.90-1.14) | 0.789 | 53.2 | 0.019 |
|  | Veneto Region 2014 | 1.06 (0.92-1.22) | 0.404 | 57.7 | 0.009 |
|  | Gini 2016 | 1.01 (0.89-1.13) | 0.928 | 51.1 | 0.025 |
|  | Clalit Health Care Services 2013 | 1.04 (0.91-1.18) | 0.564 | 59.5 | 0.006 |
|  | Danish Central Hospital Discharge Register 1997 | 1.05 (0.91-1.21) | 0.478 | 59.3 | 0.006 |
|  | D2C cohort 2011 | 1.03 (0.91-1.17) | 0.654 | 58.8 | 0.007 |
|  | Diabetes Registry Tyrol 2014 | 1.04 (0.91-1.18) | 0.573 | 59.6 | 0.006 |
|  | Xu 2015 | 1.04 (0.92-1.18) | 0.497 | 58.5 | 0.007 |
|  | Nationwide Cohort Study in Sweden 1995 | 1.04 (0.90-1.20) | 0.634 | 58.3 | 0.008 |
|  | Wang 2015 | 0.97 (0.88-1.07) | 0.563 | 25.8 | 0.198 |
|  | Zhang 2012 | 1.04 (0.92-1.18) | 0.555 | 59.5 | 0.006 |
|  | National Health Insurance Program 2014 | 1.07 (0.94-1.22) | 0.306 | 40.9 | 0.076 |
| RR/OR/HR | Uppsala Health Care Region 1991 | 0.96 (0.85-1.07) | 0.442 | 0.0 | 0.993 |
|  | Takayama Study cohort 2013 | 0.97 (0.87-1.08) | 0.569 | 0.0 | 0.986 |
|  | Ragozzino 1982 | 0.97 (0.87-1.08) | 0.554 | 0.0 | 0.987 |
|  | PHARMO Database 2017 | 0.97 (0.87-1.08) | 0.583 | 0.0 | 0.987 |
|  | Korean Cancer Prevention Study 2005 | 0.97 (0.86-1.08) | 0.546 | 0.0 | 0.987 |
|  | Clalit Health Services 2016 | 0.98 (0.85-1.12) | 0.735 | 0.0 | 0.987 |
|  | Maccabi Healthcare Services 2010 | 0.96 (0.86-1.07) | 0.486 | 0.0 | 0.995 |
|  | Japan Public Health Center- Based Prospective Study 2006 | 0.97 (0.87-1.08) | 0.606 | 0.0 | 0.991 |
|  | Cancer Prevention Study II 2004 | 0.98 (0.86-1.13) | 0.825 | 0.0 | 0.990 |
|  | Japan Collaborative Cohort Study 2006 | 0.97 (0.87-1.08) | 0.601 | 0.0 | 0.991 |
|  | Zhou 2010 | 0.96 (0.86-1.08) | 0.517 | 0.0 | 0.992 |
|  | EPOCH-JAPAN 2017 | 0.97 (0.87-1.08) | 0.603 | 0.0 | 0.994 |
