# Supplementary material for: Diabetes mellitus and the risk of gastrointestinal cancer in women compared with men: a meta-analysis of cohort studies
Source: BMC Cancer. 2018 Apr 16;18:422. doi: 10.1186/s12885-018-4351-4 (PMC5902961; doi:10.1186/s12885-018-4351-4)
Supplement: Supplementary file 6 — The findings of meta-regression for gastrointestinal cancer based on publication year, sample size, mean age, percentage of smoker, and follow-up duration. (DOC 36 kb) [file 12885_2018_4351_MOESM6_ESM.doc]

Table S1. The findings of meta-regression for gastrointestinal cancer based on publication year, sample size, mean age, percentage of smoker, and follow-up duration

| Outcomes | Effect estimate | Publication year | Sample size | Mean age | Percentage of smoker | Follow-up duration |
| --- | --- | --- | --- | --- | --- | --- |
| Esophagus cancer | SIR/SMR | 0.100 | 0.930 | 0.167 | - | 0.909 |
|  | RR/OR/HR | 0.284 | 0.416 | 0.872 | 0.749 | 0.385 |
| Gastric cancer | SIR/SMR | **0.054** | 0.860 | **0.046** | - | 0.688 |
|  | RR/OR/HR | 0.719 | 0.772 | 0.589 | 0.865 | 0.287 |
| Colorectal cancer | SIR/SMR | 0.189 | 0.226 | 0.159 | 0.192 | 0.437 |
|  | RR/OR/HR | 0.649 | 0.312 | 0.536 | 0.679 | 0.273 |
| Colon cancer | SIR/SMR | 0.809 | 0.733 | 0.701 | - | 0.895 |
|  | RR/OR/HR | 0.793 | 0.604 | 0.987 | 0.156 | 0.284 |
| Rectal cancer | SIR/SMR | 0.991 | 0.298 | 0.992 | - | 0.244 |
|  | RR/OR/HR | **0.049** | 0.418 | 0.329 | 0.276 | **0.059** |
| Hepatocellular carcinoma | SIR/SMR | **0.062** | 0.713 | 0.132 | - | 0.384 |
|  | RR/OR/HR | **0.069** | 0.562 | 0.592 | 0.815 | 0.105 |
| Pancreatic cancer | SIR/SMR | 0.914 | 0.266 | 0.522 | 0.416 | 0.930 |
|  | RR/OR/HR | 0.617 | 0.675 | 0.959 | 0.920 | 0.854 |
