# Supplementary material for: Diabetes mellitus and the risk of gastrointestinal cancer in women compared with men: a meta-analysis of cohort studies
Source: BMC Cancer. 2018 Apr 16;18:422. doi: 10.1186/s12885-018-4351-4 (PMC5902961; doi:10.1186/s12885-018-4351-4)
Supplement: Supplementary file 7 — Subgroup analyses for the relationship between diabetes mellitus and gastrointestinal cancer in women compared with men. (DOC 643 kb) [file 12885_2018_4351_MOESM7_ESM.doc]

Table S1. Subgroup analysis for the relationship between DM and esophagus cancer in women compared with men

| Effect estimate | Factor | Groups | Number of studies | RRR and 95% CI | P value | Heterogeneity (%) | P value for heterogeneity |
| --- | --- | --- | --- | --- | --- | --- | --- |
| SIR/SMR | Publication year | 2010 or after | 4 | **1.26 (1.13-1.39)** | **<0.001** | **0.0** | **0.724** |
|  |  | Before 2010 | 2 | 0.79 (0.52-1.22) | 0.294 | 0.0 | 0.615 |
|  | Country | Eastern | 3 | **1.25 (1.12-1.39)** | **<0.001** | **0.0** | **0.679** |
|  |  | Western | 3 | 1.07 (0.63-1.81) | 0.798 | 47.6 | 0.148 |
|  | Study design | Prospective | 4 | 1.19 (0.83-1.70) | 0.338 | 47.0 | 0.130 |
|  |  | Retrospective | 2 | **1.23 (1.10-1.37)** | **<0.001** | **0.0** | **0.793** |
|  | Sample size | ≥ 100,000 | 4 | 1.21 (0.98-1.50) | 0.070 | 46.9 | 0.130 |
|  |  | < 100,000 | 2 | 1.27 (0.37-4.83) | 0.722 | 0.0 | 0.787 |
|  | Mean age (years) | ≥ 60.0 | 3 | 0.81 (0.53-1.24) | 0.329 | 0.0 | 0.754 |
|  |  | <60.0 | 1 | **1.41 (1.04-1.93)** | **0.029** | **-** | **-** |
|  | DM types | II | 5 | **1.26 (1.13-1.39)** | **<0.001** | **0.0** | **0.856** |
|  |  | Both | 1 | 0.77 (0.49-1.20) | 0.251 | - | - |
|  | Follow-up duration (years) | ≥ 10.0 | 2 | **1.23 (1.10-1.37)** | **<0.001** | **0.0** | **0.937** |
|  |  | <10.0 | 4 | 1.20 (0.83-1.73) | 0.334 | 47.6 | 0.126 |
|  | Adjusted BMI | Yes | 1 | 1.15 (0.25-5.24) | 0.853 | - | - |
|  |  | No | 5 | **1.22 (1.01-1.47)** | **0.035** | **30.1** | **0.221** |
|  | Adjusted smoking | Yes | 1 | 1.15 (0.25-5.24) | 0.853 | - | - |
|  |  | No | 5 | **1.22 (1.01-1.47)** | **0.035** | **30.1** | **0.221** |
|  | Adjusted alcohol intake | Yes | 0 | - | - | - | - |
|  |  | No | 6 | **1.22 (1.06-1.42)** | **0.007** | **12.7** | **0.334** |
|  | Adjusted physical activity | Yes | 0 | - | - | - | - |
|  |  | No | 6 | **1.22 (1.06-1.42)** | **0.007** | **12.7** | **0.334** |
| RR/OR/HR | Publication year | 2010 or after | 3 | 1.05 (0.53-2.08) | 0.898 | 0.0 | 0.446 |
|  |  | Before 2010 | 2 | 0.80 (0.37-1.72) | 0.569 | 63.2 | 0.099 |
|  | Country | Eastern | 1 | 2.51 (0.53-11.86) | 0.246 | - | - |
|  |  | Western | 4 | 0.90 (0.63-1.28) | 0.547 | 0.0 | 0.417 |
|  | Study design | Prospective | 4 | 0.90 (0.63-1.28) | 0.547 | 0.0 | 0.417 |
|  |  | Retrospective | 1 | 2.51 (0.53-11.86) | 0.246 | - | - |
|  | Sample size | ≥ 100,000 | 3 | 1.09 (0.73-1.64) | 0.660 | 0.0 | 0.446 |
|  |  | < 100,000 | 2 | 0.61 (0.30-1.22) | 0.160 | 0.0 | 0.380 |
|  | Mean age (years) | ≥ 60.0 | 3 | 1.05 (0.53-2.08) | 0.898 | 0.0 | 0.446 |
|  |  | <60.0 | 1 | 1.11 (0.69-1.76) | 0.674 | - | - |
|  | DM types | II | 1 | 1.00 (0.27-3.72) | 1.000 | - | - |
|  |  | Both | 4 | 0.91 (0.55-1.50) | 0.708 | 32.4 | 0.218 |
|  | Follow-up duration (years) | ≥ 10.0 | 3 | 0.87 (0.52-1.45) | 0.586 | 26.9 | 0.255 |
|  |  | <10.0 | 2 | 1.18 (0.39-3.55) | 0.771 | 37.8 | 0.205 |
|  | Adjusted BMI | Yes | 2 | 1.18 (0.76-1.85) | 0.461 | 0.0 | 0.322 |
|  |  | No | 3 | 0.66 (0.38-1.16) | 0.148 | 0.0 | 0.626 |
|  | Adjusted smoking | Yes | 2 | 1.03 (0.68-1.57) | 0.887 | 0.0 | 0.508 |
|  |  | No | 3 | 0.89 (0.36-2.20) | 0.807 | 41.9 | 0.179 |
|  | Adjusted alcohol intake | Yes | 2 | 1.03 (0.68-1.57) | 0.887 | 0.0 | 0.508 |
|  |  | No | 3 | 0.89 (0.36-2.20) | 0.807 | 41.9 | 0.179 |
|  | Adjusted physical activity | Yes | 2 | 1.03 (0.68-1.57) | 0.887 | 0.0 | 0.508 |
|  |  | No | 3 | 0.89 (0.36-2.20) | 0.807 | 41.9 | 0.179 |

Table S2. Subgroup analysis for the relationship between DM and gastric cancer in women compared with men

| Effect estimate | Factor | Groups | Number of studies | RRR and 95% CI | P value | Heterogeneity (%) | P value for heterogeneity |
| --- | --- | --- | --- | --- | --- | --- | --- |
| SIR/SMR | Publication year | 2010 or after | 6 | **1.22 (1.09-1.37)** | **<0.001** | **33.2** | **0.187** |
|  |  | Before 2010 | 3 | 0.94 (0.79-1.12) | 0.498 | 0.0 | 0.913 |
|  | Country | Eastern | 4 | **1.23 (1.07-1.42)** | **0.003** | **51.9** | **0.101** |
|  |  | Western | 5 | 1.00 (0.86-1.15) | 0.961 | 0.0 | 0.606 |
|  | Study design | Prospective | 6 | 1.12 (0.91-1.38) | 0.281 | 63.4 | 0.018 |
|  |  | Retrospective | 3 | **1.15 (1.08-1.23)** | **<0.001** | **0.0** | **0.898** |
|  | Sample size | ≥ 100,000 | 5 | 1.13 (0.98-1.31) | 0.082 | 68.1 | 0.014 |
|  |  | < 100,000 | 4 | 1.14 (0.86-1.50) | 0.362 | 0.0 | 0.690 |
|  | Mean age (years) | ≥ 60.0 | 3 | 0.95 (0.78-1.15) | 0.592 | 0.0 | 0.661 |
|  |  | <60.0 | 3 | **1.35 (1.13-1.62)** | **0.001** | **12.7** | **0.318** |
|  | DM types | II | 6 | **1.21 (1.09-1.34)** | **<0.001** | **27.9** | **0.226** |
|  |  | Both | 3 | 0.96 (0.80-1.16) | 0.691 | 4.4 | 0.351 |
|  | Follow-up duration (years) | ≥ 10.0 | 2 | **1.15 (1.08-1.24)** | **<0.001** | **0.0** | **0.621** |
|  |  | <10.0 | 7 | 1.13 (0.95-1.36) | 0.171 | 56.0 | 0.034 |
|  | Adjusted BMI | Yes | 1 | 0.99 (0.54-1.82) | 0.978 | - | - |
|  |  | No | 8 | **1.15 (1.02-1.29)** | **0.024** | **49.2** | **0.055** |
|  | Adjusted smoking | Yes | 1 | 0.99 (0.54-1.82) | 0.978 | - | - |
|  |  | No | 8 | **1.15 (1.02-1.29)** | **0.024** | **49.2** | **0.055** |
|  | Adjusted alcohol intake | Yes | 0 | - | - | - | - |
|  |  | No | 9 | **1.14 (1.02-1.28)** | **0.020** | **43.1** | **0.080** |
|  | Adjusted physical activity | Yes | 0 | - | - | - | - |
|  |  | No | 9 | **1.14 (1.02-1.28)** | **0.020** | **43.1** | **0.080** |
| RR/OR/HR | Publication year | 2010 or after | 6 | 1.14 (0.98-1.13) | 0.100 | 0.0 | 0.423 |
|  |  | Before 2010 | 6 | 1.07 (0.91-1.25) | 0.431 | 0.0 | 0.642 |
|  | Country | Eastern | 7 | 1.07 (0.92-1.25) | 0.374 | 15.1 | 0.315 |
|  |  | Western | 5 | 1.20 (0.96-1.49) | 0.114 | 0.0 | 0.922 |
|  | Study design | Prospective | 11 | 1.12 (1.00-1.25) | 0.057 | 0.0 | 0.733 |
|  |  | Retrospective | 1 | 0.69 (0.34-1.39) | 0.298 | - | - |
|  | Sample size | ≥ 100,000 | 6 | 1.07 (0.94-1.21) | 0.317 | 0.0 | 0.591 |
|  |  | < 100,000 | 6 | 1.23 (0.98-1.55) | 0.078 | 0.0 | 0.576 |
|  | Mean age (years) | ≥ 60.0 | 3 | 0.98 (0.58-1.68) | 0.951 | 14.3 | 0.311 |
|  |  | <60.0 | 7 | 1.11 (0.98-1.25) | 0.101 | 0.7 | 0.419 |
|  | DM types | II | 4 | 1.07 (0.93-1.23) | 0.354 | 0.0 | 0.493 |
|  |  | Both | 8 | 1.17 (0.97-1.40) | 0.103 | 0.0 | 0.574 |
|  | Follow-up duration (years) | ≥ 10.0 | 7 | **1.13 (1.00-1.27)** | **0.048** | **0.0** | **0.432** |
|  |  | <10.0 | 4 | 0.80 (0.51-1.25) | 0.324 | 0.0 | 0.884 |
|  | Adjusted BMI | Yes | 6 | 1.17 (0.93-1.47) | 0.169 | 11.3 | 0.343 |
|  |  | No | 6 | 1.07 (0.94-1.22) | 0.290 | 0.0 | 0.780 |
|  | Adjusted smoking | Yes | 7 | 1.09 (0.93-1.28) | 0.270 | 0.1 | 0.422 |
|  |  | No | 5 | 1.11 (0.95-1.29) | 0.185 | 0.0 | 0.617 |
|  | Adjusted alcohol intake | Yes | 7 | 1.09 (0.93-1.28) | 0.270 | 0.1 | 0.422 |
|  |  | No | 5 | 1.11 (0.95-1.29) | 0.185 | 0.0 | 0.617 |
|  | Adjusted physical activity | Yes | 6 | 1.23 (0.99-1.52) | 0.056 | 0.0 | 0.651 |
|  |  | No | 6 | 1.06 (0.93-1.20) | 0.398 | 0.0 | 0.556 |

Table S3. Subgroup analysis for the relationship between DM and colorectal cancer in women compared with men

| Effect estimate | Factor | Groups | Number of studies | RRR and 95% CI | P value | Heterogeneity (%) | P value for heterogeneity |
| --- | --- | --- | --- | --- | --- | --- | --- |
| SIR/SMR | Publication year | 2010 or after | 7 | 0.97 (0.93-1.01) | 0.123 | 0.0 | 0.662 |
|  |  | Before 2010 | 2 | **0.64 (0.43-0.96)** | **0.030** | **0.0** | **0.867** |
|  | Country | Eastern | 3 | 0.99 (0.85-1.14) | 0.848 | 20.9 | 0.282 |
|  |  | Western | 6 | **0.88 (0.77-0.99)** | **0.037** | **0.0** | **0.694** |
|  | Study design | Prospective | 4 | 0.90 (0.78-1.05) | 0.171 | 0.0 | 0.652 |
|  |  | Retrospective | 5 | 0.94 (0.82-1.07) | 0.358 | 29.2 | 0.227 |
|  | Sample size | ≥ 100,000 | 2 | 0.97 (0.93-1.01) | 0.180 | 0.0 | 0.593 |
|  |  | < 100,000 | 7 | 0.88 (0.76-1.02) | 0.096 | 3.8 | 0.397 |
|  | Mean age (years) | ≥ 60.0 | 5 | **0.83 (0.70-0.98)** | **0.026** | **0.0** | **0.635** |
|  |  | <60.0 | 2 | 1.06 (0.74-1.53) | 0.751 | 29.5 | 0.234 |
|  | DM types | II | 8 | 0.95 (0.88-1.02) | 0.153 | 11.3 | 0.342 |
|  |  | Both | 1 | 0.85 (0.52-1.39) | 0.512 | - | - |
|  | Follow-up duration (years) | ≥ 10.0 | 4 | 0.92 (0.73-1.17) | 0.501 | 47.7 | 0.125 |
|  |  | <10.0 | 5 | 0.90 (0.79-1.02) | 0.087 | 0.0 | 0.920 |
|  | Adjusted BMI | Yes | 2 | 0.84 (0.58-1.21) | 0.349 | 27.3 | 0.241 |
|  |  | No | 7 | 0.96 (0.92-1.01) | 0.146 | 2.6 | 0.405 |
|  | Adjusted smoking | Yes | 1 | 0.66 (0.39-1.12) | 0.123 | - | - |
|  |  | No | 8 | 0.97 (0.93-1.01) | 0.101 | 0.0 | 0.521 |
|  | Adjusted alcohol intake | Yes | 0 | - | - | - | - |
|  |  | No | 9 | 0.96 (0.91-1.01) | 0.107 | 2.0 | 0.418 |
|  | Adjusted physical activity | Yes | 0 | - | - | - | - |
|  |  | No | 9 | 0.96 (0.91-1.01) | 0.107 | 2.0 | 0.418 |
| RR/OR/HR | Publication year | 2010 or after | 4 | 1.05 (0.98-1.13) | 0.163 | 0.0 | 0.649 |
|  |  | Before 2010 | 9 | 0.97 (0.79-1.18) | 0.737 | 5.0 | 0.393 |
|  | Country | Eastern | 4 | 1.01 (0.93-1.10) | 0.772 | 0.0 | 0.715 |
|  |  | Western | 9 | 1.10 (0.98-1.24) | 0.113 | 1.2 | 0.424 |
|  | Study design | Prospective | 12 | 1.04 (0.96-1.11) | 0.335 | 0.0 | 0.480 |
|  |  | Retrospective | 1 | 1.10 (0.88-1.38) | 0.408 | - | - |
|  | Sample size | ≥ 100,000 | 6 | 1.04 (0.97-1.11) | 0.278 | 0.0 | 0.642 |
|  |  | < 100,000 | 7 | 1.08 (0.76-1.53) | 0.663 | 19.0 | 0.285 |
|  | Mean age (years) | ≥ 60.0 | 2 | 1.09 (0.79-1.50) | 0.610 | 0.0 | 0.378 |
|  |  | <60.0 | 9 | 1.04 (0.93-1.16) | 0.477 | 14.8 | 0.311 |
|  | DM types | II | 3 | 1.02 (0.94-1.11) | 0.654 | 0.0 | 0.559 |
|  |  | Both | 10 | 1.09 (0.97-1.23) | 0.159 | 0.0 | 0.453 |
|  | Follow-up duration (years) | ≥ 10.0 | 9 | 1.05 (0.97-1.12) | 0.233 | 2.3 | 0.416 |
|  |  | <10.0 | 4 | 0.93 (0.57-1.53) | 0.780 | 0.0 | 0.486 |
|  | Adjusted BMI | Yes | 7 | 1.08 (0.95-1.24) | 0.252 | 0.0 | 0.722 |
|  |  | No | 6 | 1.04 (0.89-1.20) | 0.634 | 25.9 | 0.240 |
|  | Adjusted smoking | Yes | 8 | 1.05 (0.93-1.19) | 0.417 | 0.0 | 0.672 |
|  |  | No | 5 | 1.07 (0.90-1.28) | 0.447 | 31.9 | 0.209 |
|  | Adjusted alcohol intake | Yes | 7 | 1.06 (0.93-1.20) | 0.386 | 0.0 | 0.738 |
|  |  | No | 6 | 1.07 (0.89-1.28) | 0.503 | 30.5 | 0.206 |
|  | Adjusted physical activity | Yes | 6 | 1.10 (0.95-1.26) | 0.203 | 0.0 | 0.782 |
|  |  | No | 7 | 1.03 (0.90-1.18) | 0.683 | 22.1 | 0.261 |

Table S4. Subgroup analysis for the relationship between DM and colon cancer in women compared with men

| Effect estimate | Factor | Groups | Number of studies | RRR and 95% CI | P value | Heterogeneity (%) | P value for heterogeneity |
| --- | --- | --- | --- | --- | --- | --- | --- |
| SIR/SMR | Publication year | 2010 or after | 3 | 0.89 (0.76-1.05) | 0.172 | 0.0 | 0.952 |
|  |  | Before 2010 | 2 | **0.87 (0.75-1.00)** | **0.049** | **0.0** | **0.355** |
|  | Country | Eastern | 3 | 0.89 (0.76-1.05) | 0.172 | 0.0 | 0.952 |
|  |  | Western | 2 | **0.87 (0.75-1.00)** | **0.049** | **0.0** | **0.355** |
|  | Study design | Prospective | 3 | **0.88 (0.79-0.99)** | **0.029** | **0.0** | **0.616** |
|  |  | Retrospective | 2 | 0.86 (0.63-1.17) | 0.333 | 0.0 | 0.897 |
|  | Sample size | ≥ 100,000 | 3 | **0.88 (0.79-0.99)** | **0.029** | **0.0** | **0.616** |
|  |  | < 100,000 | 2 | 0.86 (0.63-1.17) | 0.333 | 0.0 | 0.897 |
|  | Mean age (years) | ≥ 60.0 | 2 | **0.85 (0.73-0.98)** | **0.028** | **0.0** | **0.880** |
|  |  | <60.0 | 2 | 0.89 (0.75-1.06) | 0.188 | 0.0 | 0.754 |
|  | DM types | II | 3 | 0.89 (0.76-1.05) | 0.172 | 0.0 | 0.952 |
|  |  | Both | 2 | **0.87 (0.75-1.00)** | **0.049** | **0.0** | **0.355** |
|  | Follow-up duration (years) | ≥ 10.0 | 0 | - | - | - | - |
|  |  | <10.0 | 5 | **0.88 (0.79-0.98)** | **0.017** | **0.0** | **0.908** |
|  | Adjusted BMI | Yes | 0 | - | - | - | - |
|  |  | No | 5 | **0.88 (0.79-0.98)** | **0.017** | **0.0** | **0.908** |
|  | Adjusted smoking | Yes | 0 | - | - | - | - |
|  |  | No | 5 | **0.88 (0.79-0.98)** | **0.017** | **0.0** | **0.908** |
|  | Adjusted alcohol intake | Yes | 0 | - | - | - | - |
|  |  | No | 5 | **0.88 (0.79-0.98)** | **0.017** | **0.0** | **0.908** |
|  | Adjusted physical activity | Yes | 0 | - | - | - | - |
|  |  | No | 5 | **0.88 (0.79-0.98)** | **0.017** | **0.0** | **0.908** |
| RR/OR/HR | Publication year | 2010 or after | 5 | 1.00 (0.79-1.27) | 0.967 | 31.5 | 0.211 |
|  |  | Before 2010 | 5 | 0.96 (0.83-1.10) | 0.562 | 0.0 | 0.484 |
|  | Country | Eastern | 4 | 1.02 (0.69-1.51) | 0.916 | 30.8 | 0.228 |
|  |  | Western | 6 | 0.96 (0.85-1.08) | 0.456 | 0.0 | 0.532 |
|  | Study design | Prospective | 8 | 0.93 (0.82-1.06) | 0.308 | 0.0 | 0.562 |
|  |  | Retrospective | 2 | 1.13 (0.84-1.51) | 0.423 | 46.2 | 0.173 |
|  | Sample size | ≥ 100,000 | 4 | 1.05 (0.93-1.20) | 0.417 | 0.0 | 0.523 |
|  |  | < 100,000 | 6 | **0.80 (0.64-0.99)** | **0.040** | **0.0** | **0.772** |
|  | Mean age (years) | ≥ 60.0 | 3 | 0.98 (0.62-1.54) | 0.920 | 65.0 | 0.058 |
|  |  | <60.0 | 5 | 1.00 (0.86-1.17) | 0.975 | 0.0 | 0.648 |
|  | DM types | II | 2 | 0.76 (0.53-1.09) | 0.133 | 0.0 | 0.404 |
|  |  | Both | 8 | 1.01 (0.90-1.13) | 0.923 | 0.0 | 0.461 |
|  | Follow-up duration (years) | ≥ 10.0 | 8 | 0.95 (0.84-1.07) | 0.371 | 0.0 | 0.575 |
|  |  | <10.0 | 2 | 1.19 (0.76-1.86) | 0.456 | 22.5 | 0.256 |
|  | Adjusted BMI | Yes | 6 | 1.05 (0.91-1.20) | 0.518 | 0.0 | 0.459 |
|  |  | No | 4 | 0.88 (0.73-1.05) | 0.156 | 0.0 | 0.463 |
|  | Adjusted smoking | Yes | 5 | 1.00 (0.85-1.16) | 0.958 | 0.0 | 0.685 |
|  |  | No | 5 | 0.96 (0.77-1.21) | 0.727 | 44.2 | 0.127 |
|  | Adjusted alcohol intake | Yes | 5 | 1.00 (0.85-1.16) | 0.958 | 0.0 | 0.685 |
|  |  | No | 5 | 0.96 (0.77-1.21) | 0.727 | 44.2 | 0.127 |
|  | Adjusted physical activity | Yes | 5 | 1.00 (0.85-1.16) | 0.958 | 0.0 | 0.685 |
|  |  | No | 5 | 0.96 (0.77-1.21) | 0.727 | 44.2 | 0.127 |

Table S5. Subgroup analysis for the relationship between DM and rectal cancer in women compared with men

| Effect estimate | Factor | Groups | Number of studies | RRR and 95% CI | P value | Heterogeneity (%) | P value for heterogeneity |
| --- | --- | --- | --- | --- | --- | --- | --- |
| SIR/SMR | Publication year | 2010 or after | 3 | 0.91 (0.69-1.21) | 0.508 | 24.6 | 0.265 |
|  |  | Before 2010 | 1 | 0.91 (0.74-1.11) | 0.358 | - | - |
|  | Country | Eastern | 3 | 0.91 (0.69-1.21) | 0.508 | 24.6 | 0.265 |
|  |  | Western | 1 | 0.91 (0.74-1.11) | 0.358 | - | - |
|  | Study design | Prospective | 2 | 0.97 (0.84-1.12) | 0.652 | 0.0 | 0.394 |
|  |  | Retrospective | 2 | 0.71 (0.46-1.09) | 0.119 | 0.0 | 0.583 |
|  | Sample size | ≥ 100,000 | 2 | 0.97 (0.84-1.12) | 0.652 | 0.0 | 0.394 |
|  |  | < 100,000 | 2 | 0.71 (0.46-1.09) | 0.119 | 0.0 | 0.583 |
|  | Mean age (years) | ≥ 60.0 | 2 | 0.89 (0.73-1.09) | 0.270 | 0.0 | 0.352 |
|  |  | <60.0 | 2 | 0.94 (0.71-1.26) | 0.684 | 34.6 | 0.216 |
|  | DM types | II | 3 | 0.91 (0.69-1.21) | 0.508 | 24.6 | 0.265 |
|  |  | Both | 1 | 0.91 (0.74-1.11) | 0.358 | - | - |
|  | Follow-up duration (years) | ≥ 10.0 | 0 | - | - | - | - |
|  |  | <10.0 | 4 | 0.94 (0.82-1.08) | 0.357 | 0.0 | 0.421 |
|  | Adjusted BMI | Yes | 0 | - | - | - | - |
|  |  | No | 4 | 0.94 (0.82-1.08) | 0.357 | 0.0 | 0.421 |
|  | Adjusted smoking | Yes | 0 | - | - | - | - |
|  |  | No | 4 | 0.94 (0.82-1.08) | 0.357 | 0.0 | 0.421 |
|  | Adjusted alcohol intake | Yes | 0 | - | - | - | - |
|  |  | No | 4 | 0.94 (0.82-1.08) | 0.357 | 0.0 | 0.421 |
|  | Adjusted physical activity | Yes | 0 | - | - | - | - |
|  |  | No | 4 | 0.94 (0.82-1.08) | 0.357 | 0.0 | 0.421 |
| RR/OR/HR | Publication year | 2010 or after | 5 | 1.20 (0.90-1.61) | 0.219 | 0.0 | 0.645 |
|  |  | Before 2010 | 4 | 1.05 (0.62-1.79) | 0.845 | 64.4 | 0.038 |
|  | Country | Eastern | 4 | 1.39 (0.90-2.15) | 0.140 | 3.7 | 0.374 |
|  |  | Western | 5 | 0.95 (0.68-1.34) | 0.772 | 50.0 | 0.091 |
|  | Study design | Prospective | 7 | 1.11 (0.75-1.63) | 0.615 | 49.8 | 0.063 |
|  |  | Retrospective | 2 | 1.19 (0.84-1.68) | 0.336 | 0.0 | 0.439 |
|  | Sample size | ≥ 100,000 | 4 | 1..09 (0.77-1.53) | 0.635 | 29.2 | 0.237 |
|  |  | < 100,000 | 5 | 1.17 (0.69-1.98) | 0.553 | 56.0 | 0.059 |
|  | Mean age (years) | ≥ 60.0 | 3 | 1.07 (0.70-1.64) | 0.746 | 0.0 | 0.373 |
|  |  | <60.0 | 4 | 1.37 (0.74-2.53) | 0.317 | 49.5 | 0.115 |
|  | DM types | II | 2 | 1.29 (0.53-3.13) | 0.569 | 44.3 | 0.180 |
|  |  | Both | 7 | 1.07 (0.77-1.48) | 0.684 | 50.0 | 0.062 |
|  | Follow-up duration (years) | ≥ 10.0 | 7 | 1.06 (0.76-1.47) | 0.732 | 48.5 | 0.070 |
|  |  | <10.0 | 2 | 1.38 (0.54-3.51) | 0.494 | 50.3 | 0.156 |
|  | Adjusted BMI | Yes | 6 | 1.28 (0.84-1.93) | 0.248 | 34.4 | 0.178 |
|  |  | No | 3 | 0.93 (0.60-1.44) | 0.738 | 61.0 | 0.077 |
|  | Adjusted smoking | Yes | 5 | 1.47 (0.85-2.56) | 0.169 | 45.2 | 0.121 |
|  |  | No | 4 | 0.93 (0.67-1.29) | 0.674 | 42.5 | 0.157 |
|  | Adjusted alcohol intake | Yes | 5 | 1.47 (0.85-2.56) | 0.169 | 45.2 | 0.121 |
|  |  | No | 4 | 0.93 (0.67-1.29) | 0.674 | 42.5 | 0.157 |
|  | Adjusted physical activity | Yes | 5 | 1.47 (0.85-2.56) | 0.169 | 45.2 | 0.121 |
|  |  | No | 4 | 0.93 (0.67-1.29) | 0.674 | 42.5 | 0.157 |

Table S6. Subgroup analysis for the relationship between DM and hepatocellular carcinoma in women compared with men

| Effect estimate | Factor | Groups | Number of studies | RRR and 95% CI | P value | Heterogeneity (%) | P value for heterogeneity |
| --- | --- | --- | --- | --- | --- | --- | --- |
| SIR/SMR | Publication year | 2010 or after | 8 | 0.96 (0.84-1.10) | 0.574 | 53.3 | 0.036 |
|  |  | Before 2010 | 2 | 0.73 (0.36-1.50) | 0.395 | 82.3 | 0.017 |
|  | Country | Eastern | 4 | 1.04 (0.87-1.24) | 0.653 | 66.9 | 0.028 |
|  |  | Western | 6 | **0.75 (0.60-0.95)** | **0.017** | **40.3** | **0.137** |
|  | Study design | Prospective | 6 | 0.87 (0.62-1.22) | 0.425 | 83.8 | <0.001 |
|  |  | Retrospective | 4 | **0.96 (0.93-1.00)** | **0.033** | **0.0** | **0.919** |
|  | Sample size | ≥ 100,000 | 4 | 0.86 (0.69-1.08) | 0.198 | 90.2 | <0.001 |
|  |  | < 100,000 | 6 | 0.94 (0.73-1.20) | 0.600 | 0.0 | 0.978 |
|  | Mean age (years) | ≥ 60.0 | 5 | 0.79 (0.56-1.12) | 0.190 | 52.1 | 0.080 |
|  |  | <60.0 | 3 | **1.18 (1.03-1.36)** | **0.021** | **0.0** | **0.472** |
|  | DM types | II | 8 | 0.97 (0.85-1.11) | 0.654 | 53.8 | 0.034 |
|  |  | Both | 2 | **0.56 (0.40-0.78)** | **0.001** | **7.3** | **0.299** |
|  | Follow-up duration (years) | ≥ 10.0 | 2 | **0.96 (0.93-1.00)** | **0.043** | **0.0** | **0.635** |
|  |  | <10.0 | 8 | 0.85 (0.65-1.12) | 0.247 | 77.3 | <0.001 |
|  | Adjusted BMI | Yes | 2 | 1.10 (0.68-1.76) | 0.700 | 0.0 | 0.985 |
|  |  | No | 8 | 0.87 (0.73-1.04) | 0.125 | 77.5 | <0.001 |
|  | Adjusted smoking | Yes | 1 | 1.09 (0.65-1.86) | 0.738 | - | - |
|  |  | No | 9 | 0.88 (0.74-1.04) | 0.133 | 74.4 | <0.001 |
|  | Adjusted alcohol intake | Yes | 0 | - | - | - | - |
|  |  | No | 10 | 0.89 (0.76-1.05) | 0.162 | 71.4 | <0.001 |
|  | Adjusted physical activity | Yes | 0 | - | - | - | - |
|  |  | No | 10 | 0.89 (0.76-1.05) | 0.162 | 71.4 | <0.001 |
| RR/OR/HR | Publication year | 2010 or after | 5 | 1.10 (0.85-1.43) | 0.462 | 0.0 | 0.523 |
|  |  | Before 2010 | 7 | **0.77 (0.66-0.91)** | **0.002** | **0.0** | **0.673** |
|  | Country | Eastern | 9 | 0.95 (0.81-1.13) | 0.581 | 0.0 | 0.634 |
|  |  | Western | 3 | **0.68 (0.54-0.86)** | **0.001** | **0.0** | **0.537** |
|  | Study design | Prospective | 11 | **0.85 (0.72-1.00)** | **0.050** | **16.2** | **0.290** |
|  |  | Retrospective | 1 | 1.32 (0.41-4.22) | 0.637 | - | - |
|  | Sample size | ≥ 100,000 | 5 | 0.88 (0.67-1.15) | 0.342 | 52.4 | 0.078 |
|  |  | < 100,000 | 7 | 0.83 (0.65-1.06) | 0.128 | 0.0 | 0.674 |
|  | Mean age (years) | ≥ 60.0 | 1 | 1.32 (0.41-4.22) | 0.637 | - | - |
|  |  | <60.0 | 10 | 0.88 (0.73-1.06) | 0.165 | 17.8 | 0.279 |
|  | DM types | II | 3 | 0.96 (0.74-1.25) | 0.782 | 37.3 | 0.203 |
|  |  | Both | 9 | **0.76 (0.63-0.92)** | **0.006** | **0.0** | **0.561** |
|  | Follow-up duration (years) | ≥ 10.0 | 9 | 0.83 (0.69-1.00) | 0.055 | 28.0 | 0.195 |
|  |  | <10.0 | 2 | 1.23 (0.60-2.53) | 0.576 | 0.0 | 0.875 |
|  | Adjusted BMI | Yes | 8 | 0.80 (0.64-1.01) | 0.058 | 0.9 | 0.422 |
|  |  | No | 4 | 0.88 (0.69-1.13) | 0.327 | 39.8 | 0.173 |
|  | Adjusted smoking | Yes | 9 | **0.80 (0.67-0.95)** | **0.010** | **0.0** | **0.595** |
|  |  | No | 3 | 0.96 (0.65-1.42) | 0.830 | 55.5 | 0.106 |
|  | Adjusted alcohol intake | Yes | 8 | **0.79 (0.66-0.94)** | **0.008** | **0.0** | **0.539** |
|  |  | No | 4 | 0.97 (0.70-1.35) | 0.848 | 35.4 | 0.200 |
|  | Adjusted physical activity | Yes | 5 | **0.74 (0.55-0.99)** | **0.046** | **10.4** | **0.347** |
|  |  | No | 7 | 0.91 (0.77-1.07) | 0.238 | 0.0 | 0.436 |

Table S7. Subgroup analysis for the relationship between DM and pancreatic cancer in women compared with men

| Effect estimate | Factor | Groups | Number of studies | RRR and 95% CI | P value | Heterogeneity (%) | P value for heterogeneity |
| --- | --- | --- | --- | --- | --- | --- | --- |
| SIR/SMR | Publication year | 2010 or after | 9 | 1.03 (0.87-1.21) | 0.735 | 60.8 | 0.009 |
|  |  | Before 2010 | 3 | 1.04 (0.86-1.28) | 0.669 | 45.4 | 0.160 |
|  | Country | Eastern | 5 | 0.99 (0.76-1.28) | 0.935 | 72.9 | 0.005 |
|  |  | Western | 7 | 1.04 (0.90-1.20) | 0.578 | 36.4 | 0.151 |
|  | Study design | Prospective | 6 | 1.08 (0.88-1.32) | 0.442 | 60.7 | 0.026 |
|  |  | Retrospective | 6 | 0.99 (0.85-1.16) | 0.900 | 43.6 | 0.114 |
|  | Sample size | ≥ 100,000 | 5 | 1.00 (0.87-1.15) | 0.997 | 74.7 | 0.003 |
|  |  | < 100,000 | 7 | 1.18 (0.92-1.51) | 0.186 | 4.5 | 0.392 |
|  | Mean age (years) | ≥ 60.0 | 5 | 1.18 (0.89-1.57) | 0.254 | 43.2 | 0.133 |
|  |  | <60.0 | 4 | 1.15 (0.91-1.45) | 0.255 | 13.7 | 0.324 |
|  | DM types | II | 9 | 1.07 (0.89-1.28) | 0.483 | 66.5 | 0.002 |
|  |  | Both | 3 | 1.01 (0.89-1.14) | 0.916 | 0.0 | 0.715 |
|  | Follow-up duration (years) | ≥ 10.0 | 3 | 1.07 (0.67-1.72) | 0.774 | 53.6 | 0.116 |
|  |  | <10.0 | 9 | 1.06 (0.93-1.20) | 0.410 | 43.2 | 0.080 |
|  | Adjusted BMI | Yes | 2 | 1.61 (0.94-2.75) | 0.085 | 0.0 | 0.432 |
|  |  | No | 10 | 1.01 (0.90-1.14) | 0.853 | 56.9 | 0.013 |
|  | Adjusted smoking | Yes | 1 | 1.98 (0.93-4.18) | 0.074 | - | - |
|  |  | No | 11 | 1.02 (0.90-1.14) | 0.789 | 53.2 | 0.019 |
|  | Adjusted alcohol intake | Yes | 0 | - | - | - | - |
|  |  | No | 12 | 1.03 (0.91-1.17) | 0.596 | 55.5 | 0.010 |
|  | Adjusted physical activity | Yes | 0 | - | - | - | - |
|  |  | No | 12 | 1.03 (0.91-1.17) | 0.596 | 55.5 | 0.010 |
| RR/OR/HR | Publication year | 2010 or after | 6 | 0.97 (0.82-1.15) | 0.734 | 0.0 | 0.900 |
|  |  | Before 2010 | 6 | 0.97 (0.83-1.12) | 0.640 | 0.0 | 0.949 |
|  | Country | Eastern | 7 | 0.96 (0.82-1.12) | 0.599 | 0.0 | 0.938 |
|  |  | Western | 5 | 0.98 (0.84-1.14) | 0.775 | 0.0 | 0.917 |
|  | Study design | Prospective | 11 | 0.96 (0.86-1.07) | 0.486 | 0.0 | 0.995 |
|  |  | Retrospective | 1 | 1.29 (0.64-2.57) | 0.477 | - | - |
|  | Sample size | ≥ 100,000 | 4 | 0.96 (0.85-1.08) | 0.514 | 0.0 | 0.848 |
|  |  | < 100,000 | 8 | 1.00 (0.78-1.29) | 0.979 | 0.0 | 0.967 |
|  | Mean age (years) | ≥ 60.0 | 2 | 1.11 (0.65-1.90) | 0.693 | 0.0 | 0.522 |
|  |  | <60.0 | 8 | 0.95 (0.84-1.07) | 0.371 | 0.0 | 0.982 |
|  | DM types | II | 3 | 0.96 (0.82-1.13) | 0.623 | 0.0 | 0.963 |
|  |  | Both | 9 | 0.98 (0.84-1.13) | 0.743 | 0.0 | 0.953 |
|  | Follow-up duration (years) | ≥ 10.0 | 9 | 0.96 (0.86-1.08) | 0.509 | 0.0 | 0.987 |
|  |  | <10.0 | 3 | 1.11 (0.64-1.92) | 0.720 | 0.0 | 0.692 |
|  | Adjusted BMI | Yes | 7 | 0.95 (0.80-1.12) | 0.532 | 0.0 | 0.903 |
|  |  | No | 5 | 0.98 (0.85-1.13) | 0.818 | 0.0 | 0.975 |
|  | Adjusted smoking | Yes | 7 | 0.94 (0.80-1.10) | 0.457 | 0.0 | 0.959 |
|  |  | No | 5 | 0.99 (0.85-1.16) | 0.929 | 0.0 | 0.905 |
|  | Adjusted alcohol intake | Yes | 6 | 0.93 (0.79-1.10) | 0.392 | 0.0 | 0.959 |
|  |  | No | 6 | 1.00 (0.86-1.16) | 0.997 | 0.0 | 0.932 |
|  | Adjusted physical activity | Yes | 4 | 0.92 (0.77-1.11) | 0.386 | 0.0 | 0.937 |
|  |  | No | 8 | 1.00 (0.87-1.14) | 0.945 | 0.0 | 0.964 |
