# Supplementary material for: Diabetes mellitus and the risk of gastrointestinal cancer in women compared with men: a meta-analysis of cohort studies
Source: BMC Cancer. 2018 Apr 16;18:422. doi: 10.1186/s12885-018-4351-4 (PMC5902961; doi:10.1186/s12885-018-4351-4)
Supplement: Supplementary file 8 — Publication biases for gastrointestinal cancer. (DOC 129 kb) [file 12885_2018_4351_MOESM8_ESM.doc]

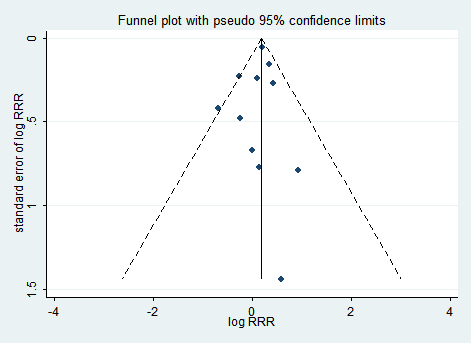


Figure S1. Funnel plot for the relationship between DM and esophagus cancer in women compared with men (P value for Egger: 0.452; P value for Begg: 0.755)


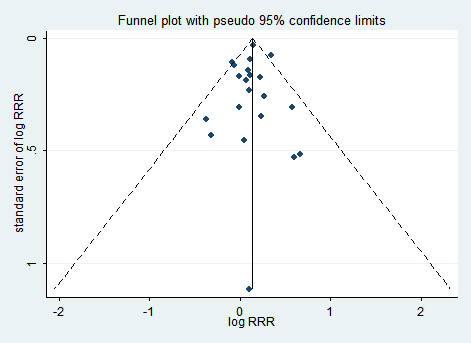


Figure S2. Funnel plot for the relationship between DM and gastric cancer in women compared with men (P value for Egger: 0.664; P value for Begg: 0.415)


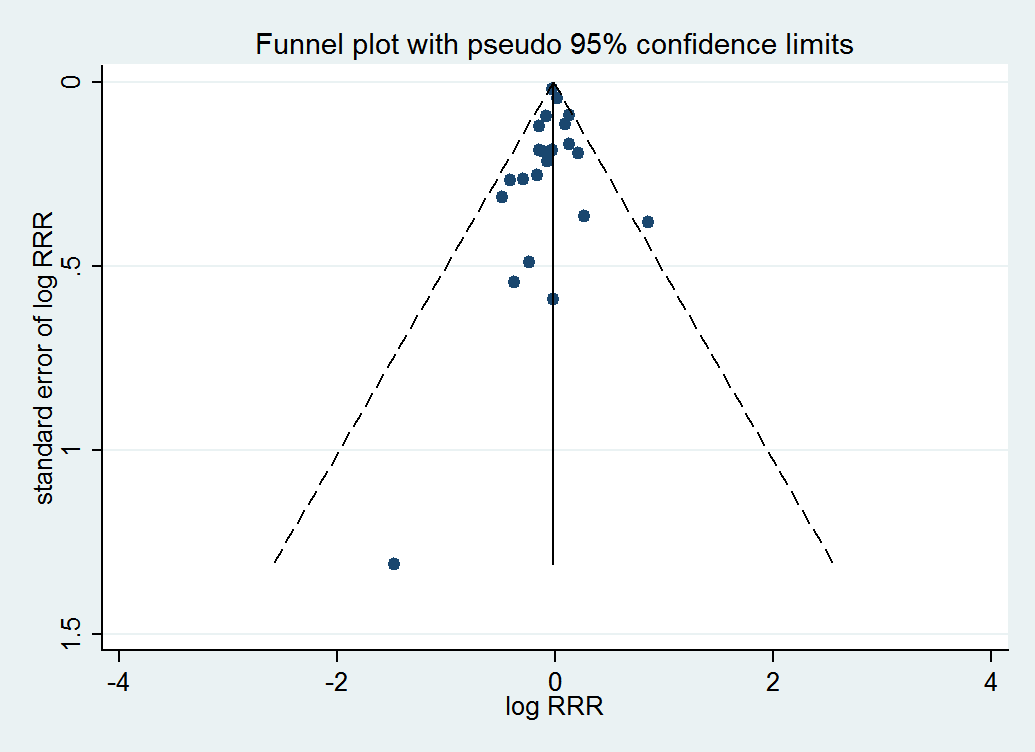


Figure S3. Funnel plot for the relationship between DM and colorectal cancer in women compared with men (P value for Egger: 0.609; P value for Begg: 0.367)


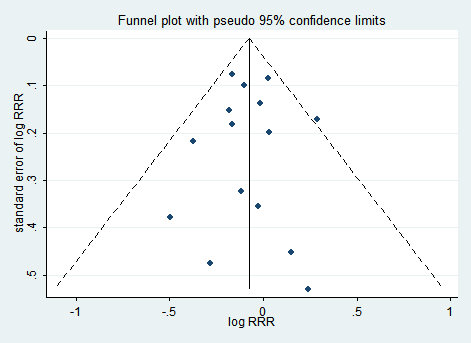


Figure S4. Funnel plot for the relationship between DM and colon cancer in women compared with men (P value for Egger: 0.982; P value for Begg: 0.767)


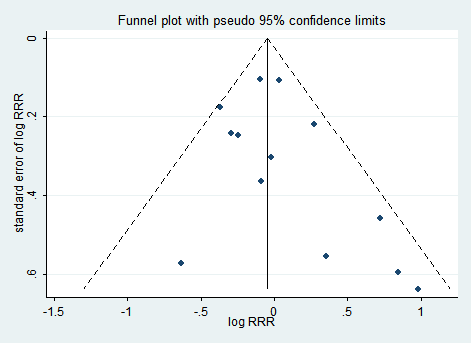


Figure S5. Funnel plot for the relationship between DM and rectal cancer in women compared with men (P value for Egger: 0.285; P value for Begg: 0.161)


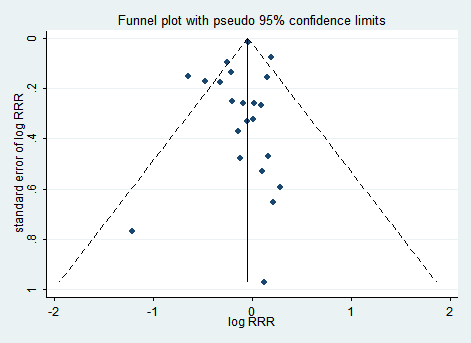


Figure S6. Funnel plot for the relationship between DM and hepatocellular carcinoma in women compared with men (P value for Egger: 0.299; P value for Begg: 0.463)


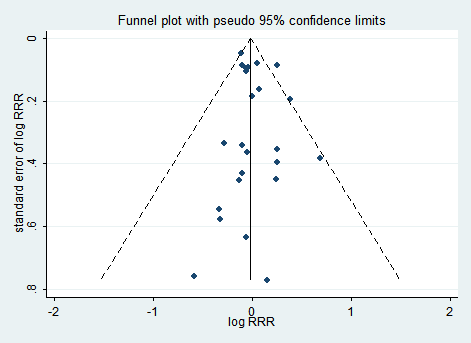


Figure S7. Funnel plot for the relationship between DM and pancreatic cancer in women compared with men (P value for Egger: 0.363; P value for Begg: 0.941)
